# Drivers of metabolic diversification: how dynamic genomic neighbourhoods generate new biosynthetic pathways in the Brassicaceae

**DOI:** 10.1111/nph.16338

**Published:** 2019-12-28

**Authors:** Zhenhua Liu, Hernando G. Suarez Duran, Yosapol Harnvanichvech, Michael J. Stephenson, M. Eric Schranz, David Nelson, Marnix H. Medema, Anne Osbourn

**Affiliations:** ^1^ Department of Metabolic Biology John Innes Centre Norwich Research Park, Colney Lane Norwich NR4 7UH UK; ^2^ Bioinformatics Group Wageningen University Droevendaalsesteeg 1 6708PB Wageningen the Netherlands; ^3^ Biosystematics Group Wageningen University Droevendaalsesteeg 1 6708PB Wageningen the Netherlands; ^4^ Department of Microbiology, Immunology and Biochemistry University of Tennessee 858 Madison Avenue, Suite G01 Memphis TN 38163 USA

**Keywords:** biosynthetic gene clusters, Brassicaceae, metabolic pathway evolution, plant interactions, specialized metabolism, terpenes

## Abstract

- Plants produce an array of specialized metabolites with important ecological functions. The mechanisms underpinning the evolution of new biosynthetic pathways are not well‐understood. Here, we exploit available genome sequence resources to investigate triterpene biosynthesis across the Brassicaceae.
- Oxidosqualene cyclases (OSCs) catalyze the first committed step in triterpene biosynthesis. Systematic analysis of 13 sequenced Brassicaceae genomes was performed to identify all OSC genes. The genome neighbourhoods (GNs) around a total of 163 OSC genes were investigated to identify Pfam domains significantly enriched in these regions. All‐vs‐all comparisons of OSC neighbourhoods and phylogenomic analysis were used to investigate the sequence similarity and evolutionary relationships of the numerous candidate triterpene biosynthetic gene clusters (BGCs) observed. Functional analysis of three representative BGCs was carried out and their triterpene pathway products were elucidated.
- Our results indicate that plant genomes are remarkably plastic, and that dynamic GNs generate new biosynthetic pathways in different Brassicaceae lineages by shuffling the genes encoding a core palette of triterpene‐diversifying enzymes, presumably in response to strong environmental selection pressure.
- These results illuminate a genomic basis for diversification of plant‐specialized metabolism through natural combinatorics of enzyme families, which can be mimicked using synthetic biology to engineer diverse bioactive molecules.

## Abstract 2

See also the Commentary on this article by Peters, **227**: 992–994.

## Introduction

Plants are chemical engineers *par excellence*, and are collectively estimated to make over a million different specialized metabolites (Afendi *et al.*, 2012). These compounds have important ecological functions, providing protection against attack by pests and pathogens, inhibiting the growth of competing plants, shaping the plant microbiome, and serving as attractants for seed dispersal agents and pollinators (Weng *et al.*, 2012a; Huang *et al.*, 2019). Plant natural products also are a rich source of bioactives for medicinal, agricultural and industrial applications (Böttger *et al.*, 2018). Despite their tremendous chemical diversity, the mechanisms underpinning the evolution of new metabolic pathways are poorly understood. Although individual enzymes are known to be recruited primarily through gene duplication, often involving sub‐ or neo‐functionalization, little is known about how pathways consisting of multiple biosynthetic steps originate (Weng, 2014). It has recently become apparent that the genes for the biosynthesis of various natural products, such as the plant defence compound avenacin A‐1 from oat (antimicrobial triterpene) (Qi *et al.*, 2004), noscapine from opium poppy (anticancer alkaloid) (Winzer *et al.*, 2012; Guo *et al.*, 2018), and dhurrin from sorghum (an insect repellent cyanogenic glycoside) (Takos *et al.*, 2011) are clustered in plant genomes. These plant biosynthetic gene clusters (BGCs) consist of at least three different classes of enzyme‐encoding genes (Medema & Osbourn, 2016) and bring unique perspectives towards metabolic innovation and diversification. First, plant BGCs have not originated from microbes via horizontal gene transfer. Instead, they appear to have arisen from plant genes by as yet unknown mechanisms, presumably honed by rounds of natural selection (Field & Osbourn, 2008). Those BGCs that make the same/very similar types of compounds are usually restricted to a narrow taxonomic window of closely related lineages (Nutzmann *et al.*, 2016), suggesting that these BGCs must have assembled relatively recently in evolutionary time. Second, plant BGCs usually contain a gene encoding an enzyme that makes the natural product scaffold, along with a combination of genes encoding other types of enzymes that modify this scaffold (tailoring enzymes) (Medema & Osbourn, 2016). The ‘rules’ that govern the evolutionary interplay between genes encoding different scaffold‐generating enzymes and tailoring enzymes are not understood. Therefore, understanding the evolution and diversification of plant BGCs is expected to offer new insights into how plants have acquired the ability to synthesize such a remarkable diversity and complexity of specialized metabolites.

The terpenoids are the major class of plant natural products, comprising ~40% of the plant natural products discovered thus far (Chassagne *et al.*, 2019); of these, the triterpenes are the largest and most structurally complex (> 20 000 reported so far) (Christianson, 2017). We have previously shown that the genes for the biosynthesis of structurally diverse triterpenes are organized in BGCs in the genome of the model plant *Arabidopsis thaliana* (Field & Osbourn, 2008). *Arabidopsis thaliana* is a member of the mustard family, Brassicaceae (Cruciferae), which includes economically important crop plants such as turnip, cabbage and oilseed rape. When we started this project, 13 high‐quality sequenced Brassicaceae genomes covering lineages I‐II (Beilstein *et al.*, 2010) and early diverging species were available (Supporting Information Table S1). The evolutionary relationships among most of these genomes are well‐defined by phylogenomic analysis (Beilstein *et al.*, 2010). Furthermore, several explicit examples of gene duplication‐promoted metabolic innovation have been reported in the Brassicaceae (Weng *et al.*, 2012b; Edger *et al.*, 2015; Liu *et al.*, 2016), demonstrating that Brassicaceae genomes are excellent working materials for exploring the genomic basis underpinning metabolic diversification. Herein we take advantage of the extensive resource of available high‐quality genome sequences to systematically investigate the genomic mechanisms underlying triterpene diversification in the wider Brassicaceae.

## Materials and Methods

### Genome mining

Genomes were retrieved from NCBI (https://www.ncbi.nlm.nih.gov/), CoGE (https://www.genomevolution.org/coge/) and phytozome v.12.0 (https://phytozome.jgi.doe.gov/) in GenBank format. To thoroughly identify *oxidosqualene cyclase *(OSC) loci, both Hmmer3 (Finn *et al.*, 2011) and Blastp (Altschul *et al.*, 1990) were used to identify OSC homologues. The Hmmer profiles (pHMMs) were downloaded from the PFAM library (Finn *et al.*, 2016). PF13243 (targeting OSC N‐terminal) and PF13249 (targeting OSC C‐terminal) were used to search for OSC homologues with hmmsearch. The cut_tc (trusted cut‐off) option was used. For Blastp, protein identity ≥ 40 and bit score ≥ 100 were used as cut‐offs, and AtCAS1 (At2g07050) was used as query sequence. Blastp identified the same OSCs present in the Hmmer analysis, but Hmmer showed slightly more candidates. The Hmmer output was aligned with outgroup protein PGGT1B_sp (geranylgeranyltransferase type I) by using Muscle (Edgar, 2004), generating a multiple sequence alignment (MSA). The MSA then was trimmed manually to keep only the conserved domains and used to build a phylogenetic tree with fasttree 2.1 using standard parameters (Price *et al.*, 2010). Support for tree nodes was assessed using fast global bootstrap iterations (Tamatakis *et al.*, 2008; Price *et al.*, 2010). Bootstrap values (1000 iterations) > 0.7 are shown in Fig. S1. Proteins grouped with outgroup PGGT1B_sp were discarded (nine proteins). To fully annotate the tree, we propagated subfamily annotations present in *A. thaliana*, resulting in three distinct groups: clade I, clade II and the sterol clade (Field and Osbourn, 2008). Another phylogeny of OSCs was reconstructed using the RAxML method (Kozlov *et al.*, 2019), which generated a tree with 134 of 164 partitions identical to the tree generated by fasttree, differing mainly in some deep ancestral splits that are difficult to resolve (Fig. S2). Pfam domains PF00067 and PF02458 were used to identify members of the cytochrome P450 (CYP) gene/protein family and the acyltransferase (ACT) gene/protein family, respectively. The identified CYP and ACT genes were pooled separately, and we used the hmmalign – trim option to trim nonconserved domains. Hmmalign‐based MSAs were taken as input to infer phylogenetic trees with fasttree. Well‐annotated *A. thaliana* CYPs (Nelson, 2009) and ACTs (Tuominen *et al.*, 2011) were used as markers to annotate protein families and subfamilies in the phylogenetic tree (Figs S3, S4). The CYPs that were functionally characterized in this study were formally assigned to subfamilies and named as CYP708A9, CYP708A10, CYP705A38, CYP708A11 and CYP705A37v2 according to procedures of the Cytochrome P450 homepage (Nelson, 2009).

### Genomic Neighbourhoods (GN) association analysis

We developed a simple tool to identify and isolate the GNs of a given list of target genes based on a user‐specified number of flanking genes. In this study, we defined ‘GNs’ as the OSC flanking regions extending five genes either side of an OSC gene. This resulted in most GNs consisting of 11 genes with two exceptions: OSCs located in a scaffold with < 11 genes result in smaller GNs, and OSCs in close proximity to each other result in overlapping GNs, which are merged into one large GN containing more than 11 genes. After identifying all *OSC* GNs across the Brassicaceae genomes, Pfam protein domain content was predicted using Hmmer.

Because of the differences in domain content among the GNs of the three OSC clades (Field & Osbourn, 2008), we explored the enrichment of protein domains in the *OSC* GNs separately for each OSC clade by comparing them to the rest of the genomes with a hypergeometric test (one‐tailed exact Fisher's test) and the Bonferroni correction for multiple comparisons. The close phylogenetic relationships between the species in our study potentially presents a problem for standard statistical procedures, because various studies have shown that the common evolutionary history of related species results in an abundance of type I errors (Martins & Garland 1991; Martins *et al.*, 2002), in part due to the nonindependence of the samples. To address the possibility of an inflation of degrees of freedom within the enrichment test, we used the OSC phylogenetic tree that we had generated previously and grouped together all monophyletic groups of leaves that share the trait targeted by the test. We used the conservative assumption that each clade of OSCs for which all GNs contain a particular protein domain represents a group of vertically inherited orthologues. Based on this, we then selected only the leaf with the highest target protein domain count for the enrichment test, instead of each leaf individually, effectively increasing the independence of the samples and resulting in a reduction of successful counts (*k* in the hypergeometric test). Because of the high abundance of CYP and Transferase domains in the *OSC* GNs, we also tested the enrichment of CYP and Transferase subfamilies separately.

Multiple methods that incorporate phylogenetic information into statistical procedures have been developed, with no method being objectively better than all others in all cases. All of them have the disadvantage, compared to the Fisher's exact test, that they do not take into account the frequency at which a given domain occurs in the rest of the genome. To further reduce the problems that arise from phylogenetic relatedness, in our study, we selected two of these phylogenetic methods to complement our initial exploration of enriched domains: phylogenetic logistic regression (PLR), and phylogenetic generalized linear mixed models (PGLMM) (Ives & Garland, 2010), both of which have been shown to be robust and adequate for studying the evolution of binary traits (Garamszegi, 2014). The main difference between the models is how the phylogenetic component is applied to the regression models: in PLR models, the dependent variable (the trait of interest) evolves to 0 or 1 through a lineage according to a switching rate, whereas PGLMM includes an additional hidden trait that evolves through the phylogenetic tree and determines the probability of the dependent variable evolving to 0 or 1 at the tip. When using regression methods such as PLR and PGLMM, the strength of association between the dependent and independent variables is not symmetrical, making their selection an important step for correct interpretation. We selected the absence and presence of OSC subfamilies in the GNs as the independent variables (i.e. the GN‐centric OSC belonging either to clade I, clade II or the sterol clade), and the absence and presence of each of the other protein domains as dependent variables; this approach allows us to interpret significant associations in the regression models that indicate whether the absence/presence of a given OSC subfamily (as opposed to others) in a GN is a strong predictor of the absence/presence of specific protein domains in their GNs.

We performed the phylogenetic regressions in R/ape v.5.0 using the ‘binaryPGLMM’ function found in the package (Paradis *et al.*, 2004) for PGLMM, and R/phylolm v.2.6 (Ho & Ané 2014) for PLR. Because we were interpreting the results of the two regression models in conjunction with other statistical tests and phylogenomic analysis, we set the significance threshold at *P* < 0.1 and did not apply multiple testing correction.

### All‐vs‐all comparison of OSC GNs

In order to assess the similarity amongst the identified *OSC* GNs, we first measured the average amino acid identity between protein domains that appear in compared GNs. In the case of GNs with multiple copies of any domain, the sequence identity of all possible domain pairs is identified and the Hungarian algorithm (Kuhn, 1955) is used to select the pairs resulting in the highest average. When a GN has extra copies of a protein domain, the highest identity scores are selected, and the additional domains are removed until both GNs have the same number of copies, after which the Hungarian algorithm is used. We next used two other tools from BiG‐Scape (Navarro‐Muñoz *et al.*, 2018) (https://git.wageningenur.nl/medema-group/BiG-SCAPE) to compare GNs. BiG‐Scape compares loci with multiple genes based on three criteria, two of which are of interest to us: domain composition similarity, measured here with the Jaccard index of identified protein domains, and domain sequence similarity (DSS), measured by averaging the sequence similarity of shared domains, with a score penalization when a GN has extra copies of a protein domain that are not present in the other. To ensure a fair comparison, only *OSC* GNs with ≥ 11 genes were selected as input for the tool.

### Mapping GNs to their whole‐genome duplication (WGD)‐derived syntenic blocks

The WGD‐derived syntenic regions in *A. thaliana* are defined by ‘anchor genes’ which retain sequence similarity with its paralogue in its sister region, and the full list of anchor genes and syntenic regions is publicly available (Freeling *et al.*, 2007). We identified the anchor genes upstream and downstream of each *OSC* GN and used them to define their corresponding sister regions. To compare each GN with its sister region, we generated a MSA with Muscle (Edgar, 2004) and a sequence similarity matrix with R/seqinr v.3.4 (Charif & Lobry, 2007).

### Ancestral states reconstruction

We pruned and isolated clade II OSCs from the phylogenetic tree and then removed the 11 leaves corresponding to GNs with < 11 genes. Given that GNs with multiple OSCs appear as multiple leaves in the phylogenetic tree, only one leaf was selected as being representative of the neighborhood based on maximizing the DSS (domain sequence similarity) index with the surrounding leaves in the tree; the nonrepresentative OSC leaves were removed.

In order to explore the evolutionary history of the selected GNs, we assigned binary traits to each leaf according to the presence (=1) or absence (=0) of the most abundant tailoring enzymes within the clade II *OSC* GNs (CYP702A, CYP705A, CYP708A and ACT IIIa) and reconstructed their ancestral states through the tree via maximum parsimony by using the mesquite software (Maddison & Maddison, 2008). We selected the maximum parsimony criterion because it does not require the assumption of an underlying model of evolution for the assembly of plant biosynthetic gene clusters (BGCs), a process that is still poorly understood.

We used the CYP and ACT phylogenetic trees to validate the ancestral state reconstruction of CYP705A and ACT IIIa in the OSC tree. For this, we pruned both trees to remove all genes in a scaffold with < 11 genes (32 CYP705As, 59 ACT IIIas) and isolated the CYP705A and ACT IIIa subtrees. Furthermore, as additional validation, we also reconstructed the ancestral states on an OSC phylogeny generated by RAxML. Although the resulting tree had some differences in the deep ancestral splits (Fig. S2), ancestral state reconstruction on this tree would lead to the same conclusions regarding multiple parallel origins of complex triterpene biosynthetic loci.

### Evolutionary tests

The OSC protein‐coding DNA sequences were aligned with translatorX (Abascal *et al.*, 2010). Genes with sequence length < 2200 nucleotides and poorly aligned genes were both filtered. The final alignment and the input tree used for the various evolutionary tests can be found on Zenodo (doi: 10.5281/zenodo.3531676). This alignment was subjected to evolutionary tests in the HyPHy package (Pond *et al.*, 2005). BUSTED analysis was used to infer whether a gene has experienced positive selection at at least one site on at least one branch (Murrell *et al.*, 2015). MEME analysis was used to detect individual sites evolving under positive selection in a proportion of branches (Murrell *et al.*, 2012). For both analyses, ‘universal genetic code’ was selected and *P* < 0.05 was set for significance.

### Plant material and growth conditions

*Capsella rubella* (Monte Gargano) and *Brassica rapa* (r‐o‐18) seeds were obtained from Lars Østergaard and *Arabidopsis lyrata* seeds (accession VLP6) from Levi Yant (John Innes Centre). Plants were grown from these seeds in a controlled growth chamber at 22°C under long‐day light conditions (16 h : 8 h, light : dark). *Nicotiana benthamiana* plants were grown in a glasshouse, under the same long‐day light conditions.

### RNA isolation and RT‐qPCR analysis

Total RNA for *A. thaliana*, *A. lyrata* and *C. rubella* were isolated from post‐flowering plants using TRIzol reagent (ThermoFisher, Carlsbad, CA, USA). Roots were dug out from soil and then washed thoroughly with water. Leaves were collected from rosettes and stems were collected from the basal second internodes. Plant material was frozen in liquid N_2_ immediately after harvesting. Reverse transcription reactions were performed using ≤ 2 μg of total RNA, random primers and a reverse transcription kit (Agilent, Santa Clara, CA, USA). The cDNAs were used as templates for quantitative (q)PCR analysis which was carried out using a CFX Real‐time PCR system (Bio‐Rad). PCR reactions (15 μl) consisted of: 7.5 μl SYBR Green I master mix solution (Roche), 1 μl gene specific primers, 5× diluted cDNAs and water. The amplification protocol involved denaturation at 95°C for 2 min, followed by 39 cycles of 95°C for 10 s and 62°C for 20 s. Amplicon dissociation curves (i.e. melting curves) were recorded after cycle 39 by heating from 65 to 95°C in 0.5°C increments. The specificity of the amplification products was verified by melting curve analysis. *ACTIN* or *PP2A* was used as a reference. Normalized gene expression levels were calculated using the 2^−ΔΔCt^ method (Livak & Schmittgen, 2001). The qPCR experiments were conducted using three independent biological replicates, each consisting of three technical replicates. Primers are listed in Table S2.

### Isolation of mutants of *C. rubella*

#### Isolation of TILLING (Targeting Induced Local Lesions IN Genomes) mutants

*Capsella rubella* TILLING mutants were ordered from RevGenUK (https://jicbio.nbi.ac.uk/revgen.html) at the John Innes Centre. Pre‐screening was carried out with DNA pools from M_2_ progeny by sequencing of PCR products amplified using specific primer pairs (Table S2). Ten M_3_ seeds from each positive line were sown. Thirteen independent lines bearing a mutation in *CYP708A9* and ten with a mutation in *CYP705A38* gene were obtained. No *CYP708A10* TILLING mutant lines were identified (Fig. S5a,b). Mutations were confirmed by sequencing. Only line Mcr705‐8 showed accumulation of tirucalla‐8,24‐dien‐3β,23‐diol (Ti2) in comparison to wild‐type (WT). This line was then crossed with the WT line. The F_2_ population derived from these crosses was used for metabolite analysis and morphological phenotyping analysis.

#### Constructs for CRISPR‐Cas9 vector and mutant isolation

Golden Gate (GG) assembly of a CRISPR‐Cas9 vector for genome editing using the seed FAST‐RFP as screen marker (Shimada *et al.*, 2010) was carried out as described recently (Castel *et al.*, 2019). Two gene‐specific guide RNA (gRNA)‐targeting sequences before a protospacer adjacent motif (PAM, NGG) site were selected (Fig. S5c). Briefly, the gRNA targeting sequences were synthesized within forward primers. Together with a universal reverse primer, a *c*. 200‐bp fragment including CRISPR‐Cas9 targeting fragment and gRNA backbone was amplified by PCR. The level 1 GG reaction contained: 20 ng pICSL90002 plasmid, 2.5 ng gRNA fused fragment, 61 ng pICH47751 (for gRNA1) or pICH47761 plasmid (for gRNA2), 1 μl Bsa I HF New England Biolabs (NEB, Ipswich, MA, USA), 1.5 μl 10× Bovine serum albumin (BSA), 1.5 μl T4 ligase buffer (10×), 1 μl T4 ligase and water (final volume 15 μl). The GG assembly protocol was as follows: (37°C for 4 min, 16°C for 3 min) × 25 cycles, then 65°C for 10 min. The gRNA targeting sequence region after GG assembly was verified by sequencing. The level 2 GG reaction contained: 160 ng pICSL4723 plasmid, 83 ng pICSL11015 plasmid, 135 ng BCJJ358 plasmid, 59 ng pICH47751 assembled gRNA1 level 1 product, 59 ng pICH47761 assembled gRNA2 level 1 product, 41 ng pICH41780, 0.5 μl Bpi I, 1.5 μl 10× BSA, 1.5 μl T4 ligase buffer (10×), 0.5 μl T4 ligase and water (final volume 15 μl). The GG assembly protocol was set up as: (37°C for 3 min, 16°C for 4 min) × 25 cycles, 65°C for 10 min. *Hin*dIII digestion was used to verify the level 2 GG assembled product. *Agrobacterium tumefaciens* strain LBA4404 carrying the CRISPR‐Cas9 construct was used for transformation of *C. rubella* plants. The floral dipping method used for *A. thaliana* (Clough & Bent, 1998) was adopted. The concentration of Silwet‐70 was reduced to 0.15%. Thirty flowering plants were used for floral dipping. T_0_ fluorescent seeds were screened with a Leica M205FA stereo microscope with a 530 nm‐red fluorescent protein (RFP) LED light source. Twelve fluorescent seeds were obtained. Two T_1_ lines that had undergone genome editing were identified. One of these (T_1_‐#20) had a 100‐bp deletion within the *CYP708A10* gene in a T_1_ leaf sample and was therefore used for next‐generation screening. Twenty‐four nonfluorescent (to avoid CRISPR/Cas9 construct on genome) T_1_‐#20 seeds were sown to generate T_2_ lines. Twelve independent T_2_ lines showed genome editing on the *CYP708A10* gene and were then sown to generate T_3_ lines. Two independent homozygous lines in the T_3_ generation were used for metabolic analyses.

### Transient expression in *N. benthamiana*

#### Construct generation

The cDNAs for the *A. lyrata AL8G20190*, *AL8G20160*, *AL8G20150* and *AL8G20140* genes were amplified from root cDNA, and those of the *C. rubella* genes *Carubv10016727m*, *Carubv10017128m*, *Carubv10017289m*, *Carubv10017243m* and *Carubv10017044m* were amplified from cDNA from flower buds. The cDNAs were cloned into the GATEWAY entry vector pDONR207 (Invitrogen) and the constructs were verified by sequencing. The corresponding genes also were amplified from genomic DNA and cloned and sequenced. *AL8G20140* and *Carubv10017243m* were misannotated in the phytozome v.12 database. The revised sequences and single nucleotide variants are listed in Table S3. The *B. rapa* genes could not be cloned from available cDNA libraries (root, leaves, stems, flowers and siliques). Genomic DNA was therefore used as template to clone the *Brara.I04560*, *Brara.I04561*, *Brara.I04562* and *Brara.I04563* genes. The sequences of these genes (if different from those represented in the phytozome v.12 database) are listed in Table S3. The cloned genes were then inserted into the pEAQ‐Dest‐1 expression vector (Sainsbury *et al.*, 2009). Constructs were verified by sequencing and introduced into *A. tumefaciens* strain LBA4404.

#### Agro‐infiltration of *N. benthamiana* leaves

The strains harbouring expression constructs were freshly grown on Lysogeny Broth (LB) plates with antibiotic selection (50 μg ml^–1^ kanamycin, 50 μg ml^–1^ rifamycin, 100 μg ml^–1^ streptomycin). For small‐scale analysis, multiple clones were picked and inoculated into 10 ml LB broth. Cultures were then incubated in a shaker (28°C and 220 rpm) for about 16 h until the OD600 reached *c*. 2.0. For large‐scale analysis, the 10 ml culture was further inoculated to make 1 L culture. LBA4404 cells were pelleted by centrifuging at 4500 ***g*** for 20 min and supernatants were discarded. The pellets were then resuspended in freshly made MMA buffer (10 mM MgCl_2_, 10 mM MES/KOH pH5.6, 150 μM acetosyringone) and diluted to OD600 0.2. For combinatorial assay, strains harbouring different constructs were mixed and infiltrated by a syringe without a needle for small‐scale analysis. For triterpene purification and NMR analysis, around 75 to 100 plants were infiltrated batch‐wise by a previously described customized vacuum infiltration system (Reed *et al.*, 2017). Leaves were harvested 6 d post‐infiltration and lyophilized.

### Metabolite extraction and analysis

#### Metabolite extraction

Three dry *N. benthamiana* leaf disks (9 cm diameter) were ground and saponified by mixture of ethanol/H_2_O/KOH pellets in 9 : 1 : 1 (v/v/w) (1 ml) at 70°C for 1 h. The ethanol was removed by evaporation (1 h, 70°C), and the samples were extracted with 1 ml ethyl acetate/H_2_O in 1 : 1 (v/v). The suspensions were centrifuged at 16 000 ***g*** for 1 min and the supernatants collected. The supernatants were dried under N_2_ and resuspended in 50 μl derivatizing reagent, 1‐(Trimethylsilyl)imidazole‐Pyridine mixture (Sigma‐Aldrich). The samples were incubated at 70°C for 30 min before analysis by GC‐MS analysis. For *C. rubella* metabolites extraction, *c*. 30 mg of fresh tissues (leaves or flowers) were ground. Samples were extracted and prepared for GC‐MS as described above. Ground samples used for LC‐MS analysis were extracted with 1 ml ethyl acetate (shaken overnight). The samples were then extracted with 500 μl of water. The supernatants were dried under N_2_ and resuspended in 100 μl methanol. The samples were all cleaned via 0.22‐μm nylon filter tube (Spin‐x centrifuge tube; Costar, Cole‐Pamer, St Neots, UK) prior to LC‐MS analysis.

#### GC‐MS and LC‐MS‐IT‐TOF analysis

GC was performed on an Agilent 7890B fitted with a Zebron ZB5‐HT Inferno capillary column (Phenomenex, Torrance, CA, USA). A 1‐μl aliquot of each sample was injected by a splitless pulse method (2.07 bar pulse pressure) with a GC inlet temperature of 250°C. The oven temperature program began from 170°C (held for 2 min) to 290°C (held for 4 min) at a speed of 6°C min^–1^ and switched to 340°C (held 1 min) at a rate of 20°C min^–1^. Helium was used as carrier gas and the flow rate was set as 1 ml min^–1^.

LC‐MS analysis was carried out on a Prominence/Nexera UHPLC system attached to an Ion‐trap time‐of‐flight (ToF) mass spectrometer (Shimadzu, Kyoto, Japan). Separation was performed on a 100 × 2.1 mm 2.6 μm Kinetex EVO reverse phase column (Phenomenemex), using the following gradient of methanol (solvent B) vs 0.1% formic acid in water (solvent A): 0 min, 70% B; 10 min, 95% B; 11 min, 95% B; 11.1 min, 70% B; 14.5 min, 70% B. The flow rate was 0.5 ml min^–1^ and the run temperature was set at 40°C. Detection was collected by positive electrospray MS. Spectra were collected from *m/z* 200–2000 with automatic sensitivity control set to a target of 70% optimal base peak intensity. Spray chamber conditions were: 250°C for curved desorbation line, 300°C for heat block, 1.3 l min^−1^ for nebulizer gas, and drying gas is ‘on’. The instrument was calibrated immediately before analysis, using sodium trifluoroacetate cluster ions according to the manufacturer's instructions.

### Triterpene purification and NMR analysis

Freeze‐dried *N. benthamiana* leaves were thoroughly extracted using a SpeedExtractor (E‐914). The extraction method was set as: solvent: ethyl acetate; pressure: 130 bar; three cycles, Hold time Cycle 1: 0 min; Hold time Cycle 2: 5 min; Hold time Cycle 3: 5 min. The extracts were dried by rotatory evaporation. The crude extracts were dissolved in ethanol and saponified by strong basic anion exchange resin amberSEP 900 hydroxide beads (Sigma‐Aldrich) for about 30 min (the solvent turned to yellow) (Stephenson *et al.*, 2018). The solvent was filtered by a mixture layers of Celite 545 (Sigma‐Aldrich) and fat‐free quartz sand (Buchi 037689). The eluted solvent was dried, loaded to an IsoleraOne system silica gel column (BioTage^®^ SNAP Vitra K‐Sil 100g, Uppsala, Sweden) and separated with a gradient of 0–100% ethyl acetate. Fractions (164 ml) were collected in 200 ml Duran bottles and aliquots (5 μl) monitored on thin layer chromatography (TLC) plate (70644‐50EA, Sigma‐Aldrich). Triterpenes were visualized by spraying of fresh‐made vanillin‐sulfuric acid reagent (1 g vanillin dissolved in 100 ml of 50% sulfuric acid.) Depending on the purity, additional separation of selected fractions was carried out using a smaller volume column (BioTage^®^ SNAP Vitra KP‐Sil 10g) with 5% ethyl acetate in dichloromethane (DCM) as eluent solvent. Fractions from each step were assessed both by TLC and GC‐MS analysis. Around 2–5 mg purified compounds were collected and subjected to NMR analysis.

Purified compounds were analysed by NMR spectroscopy. DEPT‐135, DEPT‐edited‐HSQC, COSY, and HMBC experiments were used to fully assign ^1^H and ^13^C spectra, or spectra were compared to the literature if reported previously. NMR spectra were recorded in Fourier transform mode at a nominal frequency of 400 MHz for ^1^H NMR, and 100 MHz for ^13^C NMR, using the specified deuterated solvent. Chemical shifts were recorded in ppm and referenced to the residual solvent peak or to an internal tetramethylsilane (TMS) standard. Multiplicities are described as: s, singlet; d, doublet; dd, doublet of doublets; dt, doublet of triplets; t, triplet; q, quartet; m, multiplet; br, broad; appt, apparent; coupling constants are reported in Hz as observed and not corrected for second order effects (Table S4).

## Results

### Investigation of the genome neighborhoods around triterpene synthase genes

**Figure 1:**
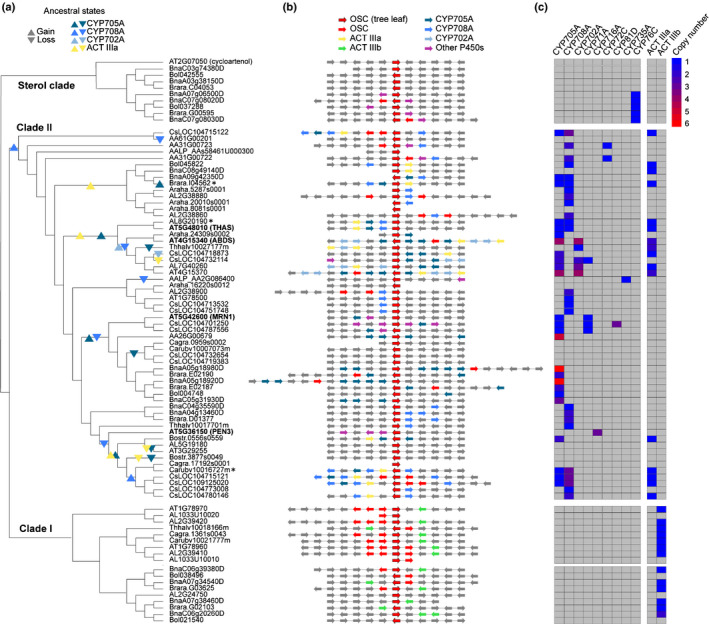
Associations between clade II oxidosqualene cyclases (OSCs) and cytochrome P450 (CYP) and acyltransferase (ACT) subfamilies in the Brassicaceae, in rapidly evolving genomic regions. (a) Maximum‐likelihood tree of clade II OSC protein sequences, including representative sterol and clade I OSCs (see Supporting Information Fig. S1 for the full 163 Brassicaceae OSCs). Characterized OSCs for *Arabidopsis thaliana* biosynthetic gene clusters (BGCs) are indicated in bold; *, OSCs characterized in this study. The ancestral states of CYPs and ACTs in the clade II OSC gene neighbourhoods (GNs) were reconstructed with maximum parsimony and inferred changes in state (gene gains and losses) are shown. (b) *OSC* GNs. The genes encoding the OSC in each tree leaf in *a* are positioned in the middle. Arrows denote the strand directionality of genes. CYP and ACT subfamilies are denoted by colours (see key). (c) Heat map showing the CYP and ACT domains in the *OSC* GNs. The colour scale bar shows copy number values.

The first committed step in triterpene biosynthesis is catalyzed by triterpene synthases – also known as OSCs. Our systematic analysis of 13 sequenced Brassicaceae genomes identified a total of 163 predicted OSC genes (Table S1). These OSC*s* grouped into three major clades: the sterol clade, containing OSC*s* implicated in primary sterol biosynthesis, and two other clades (I and II), a topology consistent with our earlier investigations of OSC*s* in *A. thaliana* (Field & Osbourn, 2008; Field *et al.*, 2011). The OSC*s* located in the previously reported *A. thaliana* BGCs all belong to clade II (Figs 1a, S1a).

We then examined the immediate GNs around all 163 OSC genes, extending five genes on either side (Fig. 1b; Table S5; all GNs are publicly available in GenBank format at doi: 10.5281/zenodo.3531676) and tested which Pfam domains were over‐represented in the *OSC* GNs relative to genome‐wide distribution. To reduce potential phylogenetic bias due to genes likely derived from common ancestors, domains that appeared consistently in the GNs of monophyletic branches of OSCs were binned and only the leaf with the largest number of domain appearances was counted (‘conservative’ hypergeometric test, *P* < 0.01; see Methods). The results indicate that the sterol, clade I and clade II OSC genes have distinct GN associations (Tables 1, S6). In general, the Pfam domains associated with sterol OSC genes do not have any anticipated roles in specialized metabolism. Although significant associations between clade I OSC genes and ACT Pfam domains were detected (Tables 1, S6), there currently is no evidence that clade I OSCs and ACTs form functional BGCs. The clade II OSC genes, however, were significantly associated with both CYP and ACT genes, both of which encode potential triterpene scaffold‐modifying enzymes (Tables 1, S6). Such associations were further supported by phylogenetic regression analyses (Table S6; Notes S1) and ‘nonconservative’ hypergeometric analysis (Table S7; Notes S1). Altogether, our analyses suggest that the evolutionary histories of clade I, clade II and sterol *OSC* GNs are distinct (Fig. 1a,b; Table 1), and importantly, that clade II OSC genes associated with CYP and ACT genes may reside in potential BGCs (Medema & Osbourn, 2016).

**Table 1:** Domains significantly associated with the Brassicaceae oxidosqualene cyclases (OSCs) from the three phylogenetic clades.

OSC	Associated Pfam domain	*P* < 0.01 (Fisher, bfr)
Sterol	NodS	3.68E‐08
Sterol	Prefoldin_2	0.00000323
Sterol	MTS*	0.000208274
Sterol	rRNA_proc.arch	0.001700795
Sterol	DSHCT	0.004179688
Clade I	ACT_IIIb*	2.05E‐17
Clade I	Fer4_7	5.86E‐16
Clade I	Fer4_9	2.53E‐15
Clade I	Fer4_4	6E‐14
Clade I	Fer4	2.14E‐12
Clade I	Fer4_10	3.56E‐12
Clade I	MOSC	0.00000399
Clade I	MOSC_N	0.00000435
Clade I	Per1	0.0000147
Clade I	EPL1	0.00049452
Clade I	Methyltransf_32*	0.001632776
Clade I	Transferase*	0.001870304
Clade I	Noc2	0.003733658
Clade I	Aminotran_5*	0.006422319
Clade I	WAK_assoc	0.006540572
Clade II	CYP705A*	1.54E‐30
Clade II	p450*	9.35E‐24
Clade II	CYP708A*	1.88E‐19
Clade II	ACT_IIIa*	1.71E‐10
Clade II	CYP702A*	2.43E‐10
Clade II	CYP716A*	0.000143077
Clade II	polyprenyl_synt*	0.002802102
Clade II	Transferase*	0.005441417

Association outcome (*P* < 0.01) of conservative Fisher's exact test (with Bonferroni correction) is listed. The full list can be found in Supporting Information Table S6. Enzymatic domains are indicated by *.

In order to further elucidate the relationship between clade II OSCs, CYPs and ACTs (in total, 58, 76 and 15 in the clade II *OSC* GNs, respectively), we identified all CYP and ACT genes in the 13 Brassicaceae and four outgroup genomes and annotated all of the CYP and ACT subfamilies by propagating the annotations of characterized genes from *A. thaliana*. Our analysis revealed that all ACT genes present in clade II *OSC* GNs belong to the ACT IIIa subfamily, whereas those in clade I *OSC* GNs belong to a distinct ACT subfamily, ACT IIIb (Fig. S3). The CYP genes associated with clade II OSC genes belong to a total of eight families, of which 86% are in the CYP708, CYP702 and CYP705 families (Figs 1c, S4).

### All‐vs‐all comparison of clade II OSC genome neighborhoods

**Figure 2:**
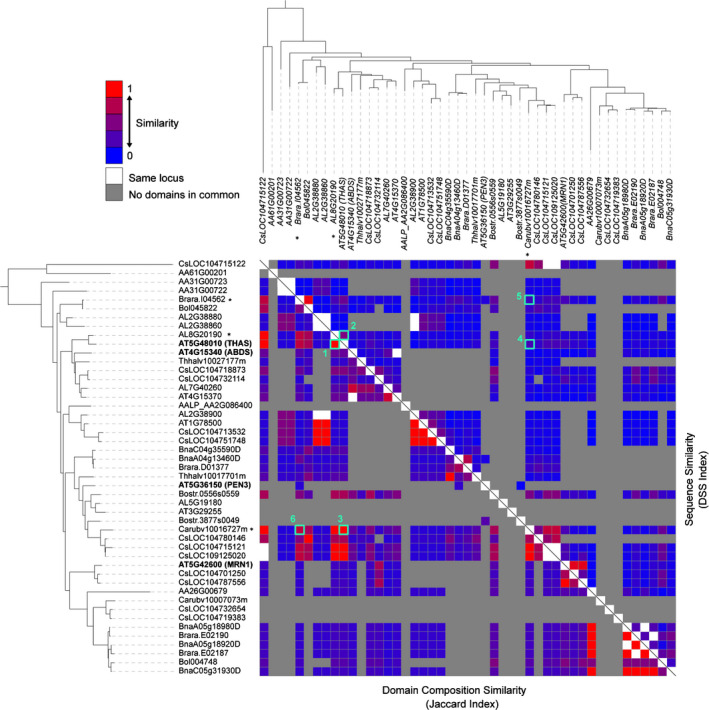
Independently evolved triterpene biosynthetic gene clusters (BGCs) frequently converge towards similar enzyme family content, yet with low mutual sequence identity. Enzymatic domains from each genome neighbourhood (GN) are compared in an all‐vs‐all fashion. The Jaccard Index was used to measure the architectural similarity (enzyme family content) across the GNs, and the domain sequence similarity (DSS) index to quantify the similarity of the underlying protein sequences. The colour scale bar shows similarity score values. The clade II oxidosqualene cyclase (OSC) tree was used to shape the structure of the heat map. Characterized OSCs for *Arabidopsis thaliana* BGCs are indicated in bold. *, OSCs characterized in this study. The diagonal line separates the Jaccard Index (left) and the DSS Index (right) comparisons. The numbers indicate: Jaccard (1) and DSS (2) index comparisons of the *A. thaliana* thalianol BGC GN with a putative BGC in *Arabidopsis lyrata*; Jaccard (3) and DSS (4) index comparisons of the *A. thaliana* thalianol BGC GN with a putative BGC in *Capsella rubella*; Jaccard (5) and DSS (6) index comparisons of the *C. rubella* BGC GN with a putative BGC in *Brassica rapa*.

Because of the abundance of genes encoding potential triterpene scaffold‐modifying enzymes in clade II *OSC* GNs, 72% (36 of 50) of which fulfil our definition of BGCs (Medema & Osbourn, 2016), we focused on this clade to explore their evolutionary relationships. After pruning the clade to ensure that all GNs had at least five genes flanking each OSC, we performed an all‐vs‐all comparison of these GNs using three distinct methods: to measure architectural similarity (enzyme family content), we used the Jaccard index of Pfam domains, and as proxies for specific enzymatic function similarity or divergence, we measured the average amino acid identity of shared protein domains and the DSS index, which further considers the differing number of appearances of each domain in each GN (see Methods) (Fig. 2; Table S8). To avoid bias due to flanking genes not involved in specialized metabolism, we took into account only those domains known to be involved in specialized metabolic pathways in plants (Kautsar *et al.*, 2017). Intriguingly, on the one hand, the domain content of clade II *OSC* GNs is very dynamic (439 of 741 GN pairs having no shared domains), suggesting rapid gene turnover in the OSC flanking regions. On the other, around 24% (73 of 302) of the GN pairs that have domains in common have highly similar domain compositions (Jaccard index ≥ 0.5), consistent with nonrandom associations of CYPs and ACTs with the OSCs from this clade. Surprisingly, in 58% of the GN pairs, the average amino acid identity between shared domains (177 of 302) falls below 50%, and 84% of GN pairs (257 of 302) have a DSS index ≤ 0.3. This suggests that OSC‐centric neighbourhoods with similar enzyme family compositions are either undergoing rapid evolution or have evolved multiple times independently. In line with the latter, ancestral state reconstruction, based on maximum parsimony and supported by detailed analysis in the phylogeny of the tailoring enzymes (Notes S1), indicates multiple parallel gene gain and loss events of the major CYPs and ACTs in the OSC‐centric neighbourhoods (Figs 1a, S6). Interestingly, three of the six clade II *OSC* GNs (including the previously characterized thalianol and marneral BGCs) in *A. thaliana* are located in dynamic chromosomal regions that do not show synteny with regions originating from the ancient WGD in Brassicaceae (Freeling *et al.*, 2007). This again indicates that the loci around clade II OSCs are highly dynamic. Thus, the evolutionary dynamics of the OSC genomic neighbourhoods indicates a general pattern of independent and parallel evolution of the enzyme family compositions of these loci.

### Functional analysis of selected representative BGCs

**Figure 3:**
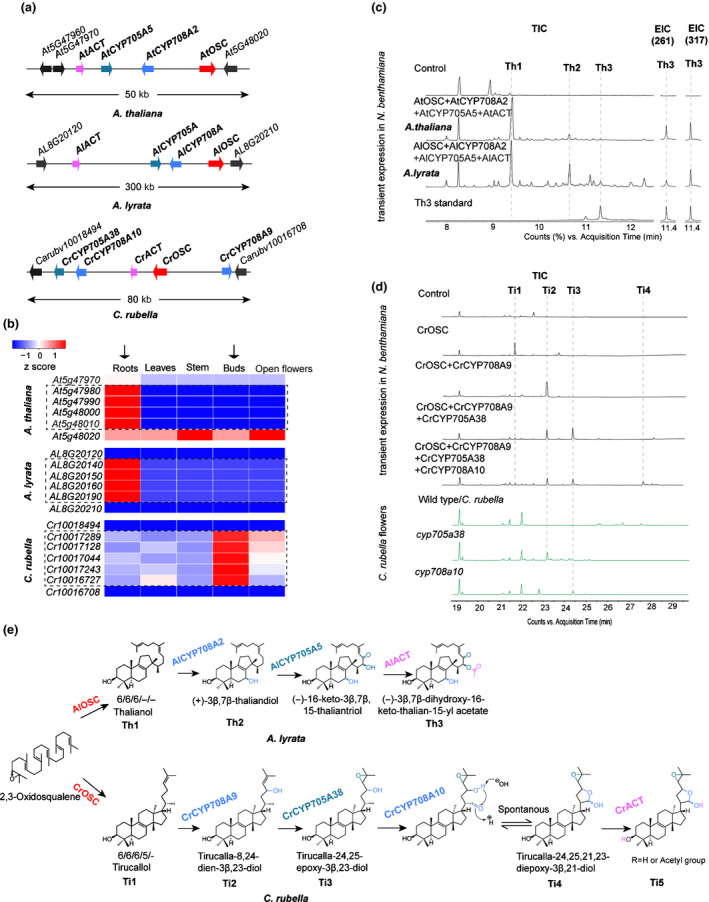
Conservation and diversification of similar triterpene biosynthetic gene clusters (BGCs). (a) Schematic of three triterpene BGCs from *Arabidopsis thaliana*, *Arabidopsis lyrata* and *Capsella rubella*. (b) Expression profiles of the BGC and BGC‐flanking genes for the three BGCs shown in (a). The numerical values for the blue‐to‐red gradient bar represent normalized expression levels relative to roots from reverse transcription quantitative (RT‐q)PCR analysis (2^−∆∆Ct^). (c) Transient expression of *A. thaliana* thalianol BGC genes and the putative *A. lyrata* BGC genes in *Nicotiana benthamiana*. GC‐MS total ion chromatograms (TICs) and extracted ion chromatograms (EICs) for two characteristic ions (261 and 317) of Th3 are shown. (d) TIC of extracts from: *N. benthamiana* leaves transiently co‐expressing different combinations of the *C. rubella* BGC genes (upper panel); flower extracts from wild‐type and mutant *C. rubella* lines (lower panel). The data are representative of at least two separate experiments. (e) The thalianol pathway in *A. lyrata* and the *C. rubella* tirucallol pathway. Enzymes are colour‐coded according to the key in (a).

In some cases, ancestral state reconstruction indicates that similar enzyme composition can be traced back to a recent common ancestry. For example, the CYP705A and ACT IIIa domains were present in some reconstructed ancestral *OSC* GNs (Fig. S6) at the base of certain monophyletic branches, including the one containing the previously characterized thalianol BGC from *A. thaliana* (Fig. 3a). The thalianol BGC consists of genes encoding an OSC, two CYPs (a CYP708A and a CYP705A), and one ACT (Field & Osbourn, 2008; Huang *et al.*, 2019). Our previous work and the current analysis indicate that a closely related (as yet uncharacterized) BGC is present in the sister species *A. lyrata* (Field *et al.*, 2011) (Fig. 3a)*.* The genes within these two BGCs (labelled 1 and 2, respectively in Fig. 2) share high nucleotide sequence identity and occur in the same genomic order. These clusters both have root‐specific expression profiles (Fig. 3b) and are located in syntenic genomic blocks (Field *et al.*, 2011) (Table S9). They are therefore likely to share a common evolutionary origin. To evaluate the function of the predicted *A. lyrata* BGC, we cloned the genes and expressed them in *N. benthamiana* using transient agro‐infiltration (Sainsbury *et al.*, 2009; Reed *et al.*, 2017). These experiments confirmed that the *A. lyrata* OSC, CYP708A, CYP705A and ACT enzymes are functionally equivalent to their counterparts in *A. thaliana*, and when co‐expressed together produce (‐)‐3β,7β‐dihydroxy‐16‐keto‐thalian‐15‐yl acetate (Th3) (Huang *et al.*, 2019) (Fig. 3c). The presence of thalianol pathway BGCs in *A. thaliana* and *A. lyrata* thus represents an example of conserved BGCs in closely related species.

By contrast, many other pairs of putative BGCs identified in our large‐scale analysis of Brassicaceae genomes appeared to have similar domain composition, but very limited sequence similarity (Fig. 2). Moreover, the ancestral state reconstruction suggested that CYPs and ACTs had been recruited to these GNs independently. An example of this is a predicted BGC in pink shepherd's purse (*C. rubella*). Just like the *A. thaliana* and *A. lyrata* thalianol clusters, this BGC contains domains for OSC, CYP708, CYP705 and ACT enzymes. However, the gene order is different, the sequence identity with the *A. thaliana* thalianol cluster is low (labelled 3 and 4, respectively in Fig. 2; average domain amino acid identity = 45% and DSS = 0.3) and there is an additional *CYP708A* gene (Fig. 3a). Unlike the thalianol BGC, the *C. rubella* gene cluster is expressed preferentially in the buds (Fig. 3b). The OSC from this cluster yielded a product (Ti1) when expressed in *N. benthamiana*, which we subsequently showed by NMR to be the triterpene tirucallol (Fig. 3d,e; Table S4A). Through combinatorial expression we then showed that the enzymes encoded by the neighbouring CYP and ACT genes were able to successively modify tirucallol. Specifically, co‐expression of the OSC with CYP708A9 yielded (Ti2); further inclusion of CYP705A38 gave conversion of (Ti2) to (Ti3); compound (Ti3) was further modified by CYP708A10 to give (Ti4) (Fig. 3d). The ACT was able to further modify (Ti4) to give (Ti5) with very low conversion (Fig. S7). The structures of compounds Ti1, Ti2, Ti3 and Ti4 were determined by NMR (Table S4B–D). That of Ti5 was inferred by LC‐MS (Fig. S7). The pathway is shown in Fig. 3e. Metabolite analysis of *C. rubella* TILLING/CRISPR‐Cas9 mutants for *CYP705A38* and *CYP708A10* (Figs 3d, S5) provided further support for the pathway shown in Fig. 3e. Phylogenetic analysis indicates that CYPs and ACTs associated with the *A. thaliana* thalianol and *C. rubella* tirucallol BGCs are scattered across their respective enzyme family trees, rather than forming a subclade (Figs S3, S4). Furthermore, the *A. thaliana *thalianol BGC and *C. rubella* tirucallol BGC can be traced back to different ancestral crucifer karyotype blocks (Lysak *et al.*, 2016) (W and K‐L, respectively; Table S9). Collectively, our results indicate that although the *C. rubella* BGC has superficial similarities with the *A. thaliana* and *A. lyrata* thalianol pathway BGCs in terms of domain composition, it is functionally distinct and has evolved independently.

We also investigated the function of a candidate BGC from *Brassica rapa*, which belongs to Brassicaceae lineage II. This group separated from common ancestors of Brassicaceae lineage I species (which include *A. thaliana* and *C. rubella*) around 20 Myr ago (Hohmann *et al.*, 2015). This candidate BGC contains genes predicted to encode an OSC, two CYPs (a CYP708A and a CYP705A), and an ACT. Expression of these genes in *N. benthamiana* revealed that the OSC produces the triterpene euphol, and that the associated CYP705A and ACT are able to metabolize this triterpene (Fig. S8). Activity was not, however, detected for the CYP708A. Interestingly, euphol is an epimer of tirucallol. Given the complex genomic history of the Brassicaceae, common ancestry cannot be fully excluded for these loci. Yet, the fact that the tirucallol and euphol OSCs are located in different ACK blocks (L and I, respectively; Table S9), the paraphyly of these two OSCs in the OSC phylogeny, and the observation that the ACTs within these loci are not monophyletic, indicate that parallel events are likely to have taken place in the evolutionary history of these two loci (labelled 5 and 6 in Fig. 2), in this case with likely a parallel metabolic outcome.

**Figure 4:**
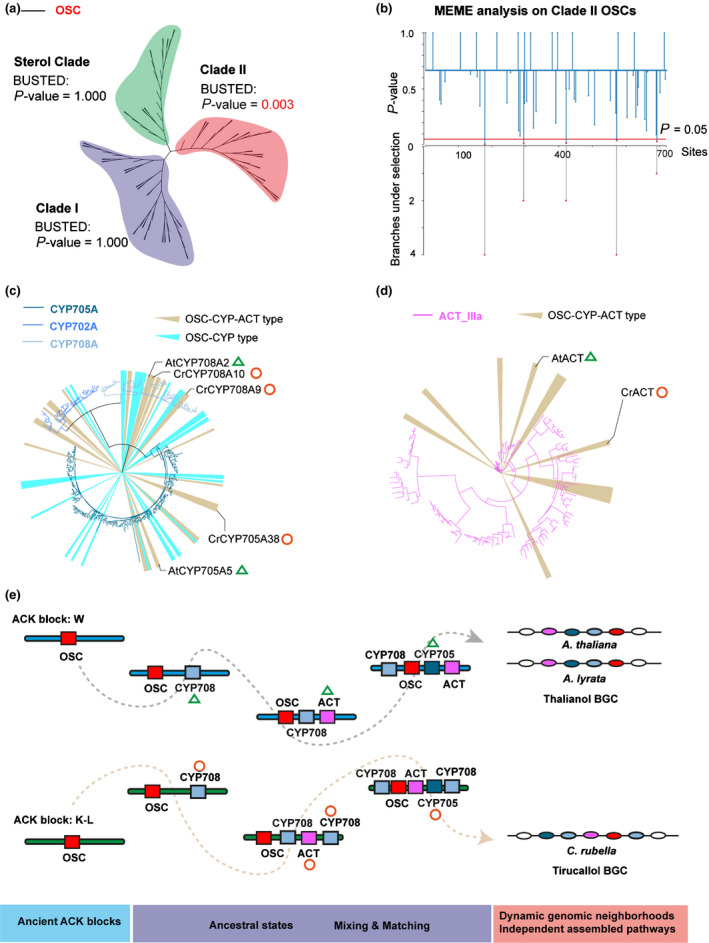
Independent evolution of clade II oxidosqualene cyclase (OSC) related biosynthetic gene clusters. (a) BUSTED (Branch‐Site Unrestricted Statistical Test for Episodic Diversification) analysis on the three clades of Brassicaceae OSCs. Evidence for gene‐wide positive selection was found for clade II OSC. (b) Mixed Effects Model of Evolution (MEME) analysis on clade II OSCs. Five sites from limited branches were found to be under positive selections (*P* < 0.05). (c, d) Clade II OSC/CYP and clade II OSC/CYP/ACT associations are marked on cytochrome P450 (CYP) (c) and acyltransferase (ACT) (d) phylogenetic trees. The genes of *Arabidopsis thaliana* thalianol BGC and *Capsella rubella* tirucallol BGC are indicated. (e) Proposed model for the evolution of the *A. thaliana* and *A. lyrata* thalianol BGCs and the *C. rubella* tirucallol BGC. The plausible ancestral states of cluster assembly were drawn based on ancestral states reconstructions. The recruited domains can be traced on the phylogeny in (c) and (d).

We next applied Branch‐Site Unrestricted Statistical Test for Episodic Diversification (BUSTED) analysis across the three OSC clades, and we found evidence for gene‐wide positive selection on clade II (Fig. 4a). In line with this, Mixed Effects Model of Evolution (MEME) analysis identified five sites in the clade II OSC alignment under positive selection (Fig. 4b). The episodic selection, however, is restricted to a limited number of branches, indicating clade II OSCs are evolving independently. Consistently, tailoring genes (CYP and ACT) contributing to clade II OSC/CYP or clade II OSC/CYP/ACT associations are scattered on their respective phylogenetic trees (Fig. 4c,d). Together with the ancestral state reconstruction, we propose that dynamic ancestral *OSC* GNs independently shuffle a core palette of decorating domains, forming divergent BGCs throughout the radiation of Brassicaceae species (Fig. 4e).

## Discussion

How individual enzymes have evolved to achieve metabolic diversity is relatively well‐understood (Benderoth *et al.*, 2006; Weng *et al.*, 2012a; Hofberger *et al.*, 2013; Hamberger & Bak, 2013; Moghe & Last, 2015; Barco & Clay, 2019), whereas the mechanisms of evolution of multi‐step pathways are more elusive. In comparison to nonclustered pathways, biosynthetic gene clusters (BGCs) provide unique material with which to systematically study the evolutionary processes underpinning the birth of plant metabolic pathways. Here, our systematic genomic neighbourhood (GN) analysis of oxidosqualene cyclases (OSCs) across multiple Brassicaceae genomes has revealed that clade II OSC genes are predisposed to clustering with genes for potential triterpene scaffold‐modifying enzymes. The number of genomes that we used in this study is small in relation to the >3000 species in the Brassicaceae. Ancestral state reconstruction with a larger dataset – once other genome sequences become available – may therefore produce a different outcome. However, our dataset is representative of the two major Brassicaceae lineages (I and II) and also includes the early diverging species *Aethionema arabicum*. The overall picture delineating parallel recruitment events in these dynamic genomic neighborhoods is highly supported and is corroborated by the evidence from cytochrome P450 (CYP) and acyltransferase (ACT) phylogenies as well as ancestral karyotype reconstruction.

Compared to previous analyses of plant BGCs (Kautsar *et al.*, 2017; Töpfer *et al.*, 2017; Schläpfer *et al.*, 2017), our phylogenomic analysis of OSCs across multiple Brassicaceae genomes provides, for the first time, a comprehensive picture of their genomic evolution across all relevant loci, whether they constitute gene clusters or not. Our phylogenetic analyses of the key gene families, along with their contextualization in whole‐genome duplication (WGD)‐derived subgenomes (Fig. S9), provide clear evidence for recurrent independent assembly of BGCs containing OSC, CYP and ACT genes across the Brassicaceae, leading to divergent or parallel metabolic outcomes (Fig. 4e).

**Figure 5:**
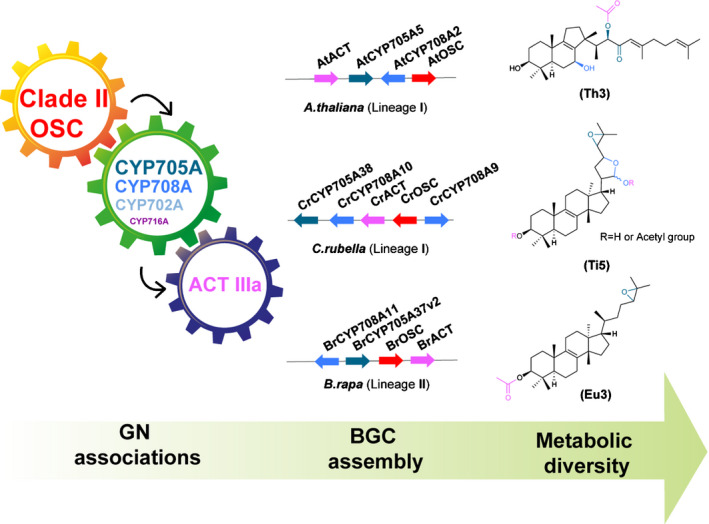
Drivers of triterpene diversification in the Brassicaceae. The biosynthesis of diverse triterpenes in the Brassicaceae is achieved by ‘mixing, matching and diverging’ a core palette of clade II oxidosqualene cyclases (OSCs), cytochrome P450s (CYPs) belonging primarily to the CYP705A, CYP708A, and CYP702A subfamilies, and acyltransferases (ACTs) belonging to the ACT IIIa subfamily. Examples of characterized biosynthetic gene clusters (BGCs) and their products are shown. Arrows denote the strand directionality of genes.

Because of the importance of triterpenes in mediating interactions with herbivores and microbiota (Hostettmann & Marston, 1995; Papadopoulou *et al.*, 1999; Nielsen *et al.*, 2010; Sohrabi *et al.*, 2015; Zhou *et al.*, 2016; Huang *et al.*, 2019), we speculate that, similar to the case of resistance gene clusters in plant genomes (Michelmore & Meyers, 1998), triterpene GNs undergo rapid and dynamic evolution, forming ‘evolutionary playgrounds’ that enable rapid adaption to ever‐changing environmental stresses (Field *et al.*, 2011). Triterpenoid scaffold diversification is achieved using a specific palette of CYPs (primarily CYP705A, CYP708A, CYP702A) and ACTs (ACT IIIa) (Fig. 5). The CYP716A family, which has been suggested as a major contributor to the diversification of triterpenoids in eudicot plants (Miettinen *et al.*, 2017), appeared only rarely among the CYPs in Brassicaceae in clade II *OSC* GNs. The molecular mechanisms by which BGCs form is not yet known, although miniature inverted‐repeat transposable elements (MITEs) have been implicated in cluster assembly and/or regulation (Boutanaev & Osbour, 2018).

Our investigations reveal a novel genomic basis for metabolic diversification in plants through mixing, matching and diverging natural combinations of enzyme families. They further open up opportunities to mimic and expand on plant metabolic diversity by using synthetic biology approaches to engineer diverse bioactive molecules through combinatorial biosynthesis (Fig. 5), for which efficient heterologous expression platforms are now available in yeast (Scheler *et al.*, 2016; Arendt *et al.*, 2017; Bathe *et al.*, 2019) as well as tobacco (Reed *et al.*, 2017). Thus, our increased understanding of pathway formation paves the path to further explore and exploit the biological activities of triterpenes and other plant natural products toward applications in medicine and agriculture.

## Author contributions

ZL, HGSD, MHM and AO conceived and designed the project; MHM and AO supervised the project; ZL carried out initial genome mining for OSC genes, performed evolutionary tests, carried out functional analysis of biosynthetic gene clusters, and isolated and characterized plant mutants; HGSD carried out genome mining, GN analysis, all‐vs‐all comparison of *OSC* GNs and ancestral state reconstruction; YH contributed to the GN analysis and the all‐vs‐all comparison of *OSC* GNs; ZL and HGSD carried out phylogenetic analysis; MJS performed all NMR analyses and structural assignments and advised on natural product purification; HGSD and MES performed syntenic block analysis; DN classified CYPs according to established convention; and ZL, HGSD, MHM and AO wrote the manuscript. ZL and HGSD contributed equally to this work.

## Supporting information

Please note: Wiley Blackwell are not responsible for the content or functionality of any Supporting Information supplied by the authors. Any queries (other than missing material) should be directed to the *New Phytologist* Central Office.

**Fig. S1** An overview of Brassicaceae OSCs: their phylogeny, genomic neighbourhoods and copy number of potential tailoring genes.
**Fig. S2** Topology comparison of clade II OSC trees generated by (a) RAxML and (b) FastTree.
**Fig. S3** Phylogeny of ACTs that are associated with clade I and clade II OSCs.
**Fig. S4** Phylogeny of CYPs that are associated with clade II OSCs.
**Fig. S5** Identification of *C. rubella* TILLING/CRISPR‐Cas9 mutants for *CYP705A38* and *CYP708A10*.
**Fig. S6** Ancestral states reconstruction of CYP and ACT subfamilies in clade II *OSC* GNs.
**Fig. S7** Functional analysis of the ACT from the *C. rubella* BGC.
**Fig. S8** Functional characterization of *B. rapa* euphol BGC.
**Fig. S9** Amino‐acid sequence similarity of proteins encoded by *A. thaliana* *OSC* GNs located within WGD‐derived syntenic block and their respective sister loci.
**Notes S1** Supplementary results.
**Table S1** 163 Brassicaceae OSCs identified in this study.
**Table S2** Primers used in this study.

**Table S3** Revised sequence for functional analysis.
**Table S4** NMR data.

**Table S5** 126 OSC‐centric GNs identified in this study.
**Table S6** GN associations with different regression analysis.

**Table S7** Fisher's exact test on individual genomes.
**Table S8** BIG‐SCAPE index values and average amino acid identity of GN pairs.
**Table S9** The relationship of OSC‐centric GNs and WGD‐derived ACK blocks.

## Data Availability

We have made Python and R scripts employed for this study available at https://bitbucket.org/herl91/genomic_neighborhoods_tools/.
