# Supplementary material for: Drivers of metabolic diversification: how dynamic genomic neighbourhoods generate new biosynthetic pathways in the Brassicaceae
Source: New Phytol. 2019 Dec 28;227(4):1109–23. doi: 10.1111/nph.16338 (PMC7383575; doi:10.1111/nph.16338)
Supplement: Supplementary file 1 — Fig. S1 An overview of Brassicaceae OSCs: their phylogeny, genomic neighbourhoods and copy number of potential tailoring genes. Fig. S2 Topology comparison of clade II OSC trees generated by (a) RAxML and (b) FastTree. Fig. S3 Phylogeny of ACTs that are associated with clade I and clade II OSCs. Fig. S4 Phylogeny of CYPs that are associated with clade II OSCs. Fig. S5 Identification of C. rubella TILLING/CRISPR‐Cas9 mutants for CYP705A38 and CYP708A10. Fig. S6 Ancestral states reconstruction of CYP and ACT subfamilies in clade II OSC GNs. Fig. S7 Functional analysis of the ACT from the C. rubella BGC. Fig. S8 Functional characterization of B. rapa euphol BGC. Fig. S9 Amino‐acid sequence similarity of proteins encoded by A. thaliana OSC GNs located within WGD‐derived syntenic block and their respective sister loci. Notes S1 Supplementary results. Table S1 163 Brassicaceae OSCs identified in this study. Table S2 Primers used in this study. [file NPH-227-1109-s001.pdf]

New Phytologist Supporting Information

Article title: Drivers of metabolic diversification: how dynamic genomic neighborhoods generate new biosynthetic pathways in the Brassicaceae

Authors: Zhenhua Liu[1]#, Hernando G. Suarez Duran[2]#, Yosapol Harnvanichvech[2], Michael J. Stephenson1, M. Eric Schranz[1], David Nelson[3], Marnix H. Medema[2]* & Anne Osbourn[1]*

Article acceptance date: 17 November 2019

The following Supporting Information is available for this article:

1. **Supplementary Figures**
2. **Supplementary Tables**
3. **Notes S1**
4. **Supplementary Reference**

1. Supplementary figures

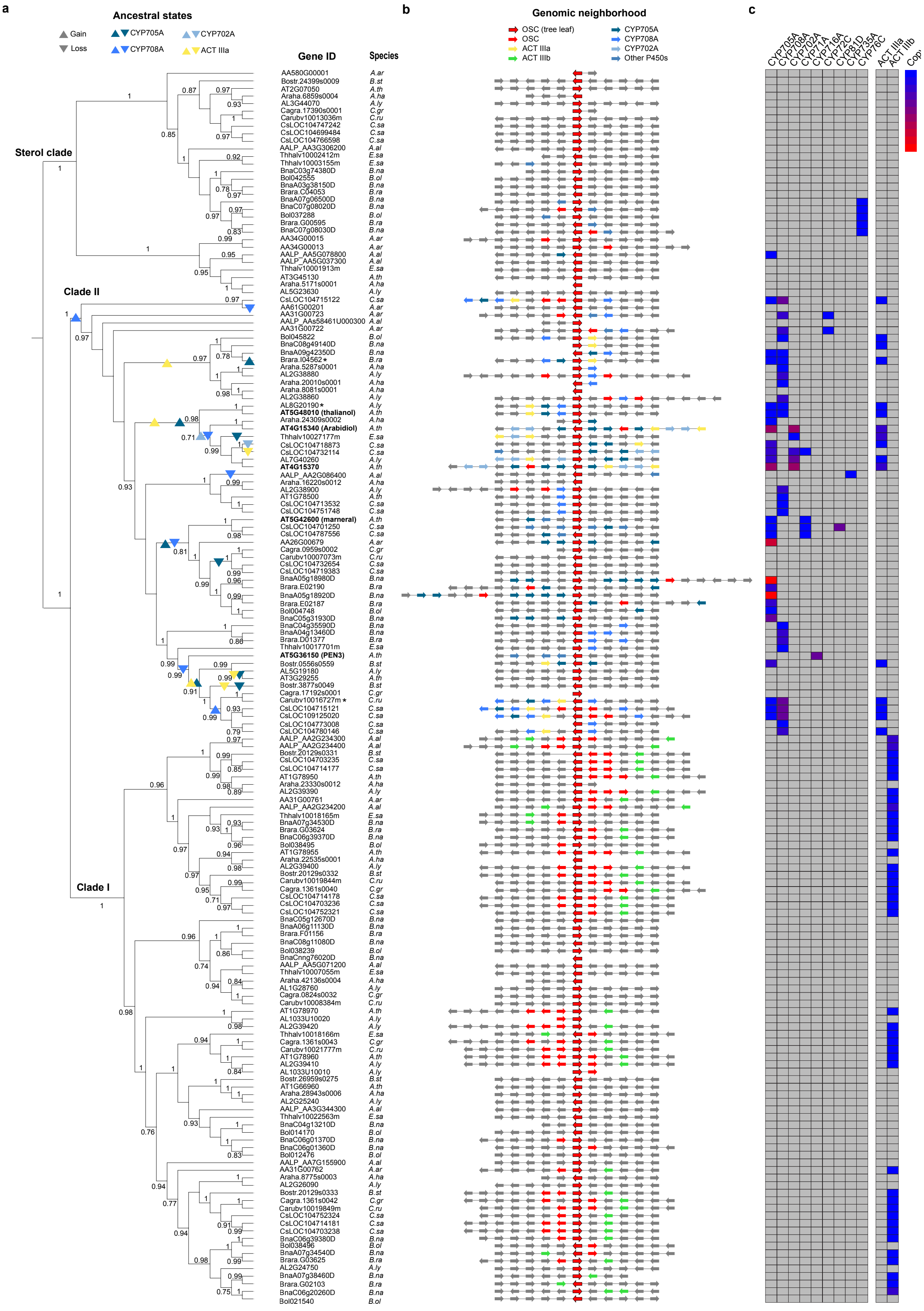
a
Ancestral states
Gain
Loss
CYP705A
CYP708A
CYP702A
ACT IIIa
Gene ID
Species
Sterol clade
Clade II
Clade I
AT5G48010 (thalianol)
AT4G15340 (Arabidiol)
AT4G15370
AT5G42600 (marneral)
AT5G36150 (PEN3)
b
Genomic neighborhood
OSC (tree leaf)
OSC
ACT IIIa
ACT IIIb
CYP705A
CYP708A
CYP702A
Other P450s
c
CYP705A
CYP708A
CYP702A
CYP71A
CYP716A
CYP72C
CYP81D
CYP735A
CYP76C
ACT IIIa
ACT IIIb

Fig. S1. An overview of Brassicaceae OSCs: their phylogeny, genomic neighbourhoods and copy number of potential tailoring genes.

a, Maximum-likelihood tree of the full 163 Brassicaceae OSC protein sequences. Characterized OSCs for *A. thaliana* BGCs are indicated in bold; *, OSCs characterized in this study. The ancestral states of CYPs and ACTs in the clade II OSC GNs were reconstructed with maximum parsimony (Notes S1) and inferred changes in state (gene gains and losses) are shown. Bootstrap values (with 1000 iterations) > 0.7 are shown. **b,** OSC GNs. The genes encoding the OSC in each tree leaf in ***a*** are positioned in the middle. Arrows denote the strand directionality of genes. CYP and ACT subfamilies are denoted by colours (see key). **c**, Heat map showing the CYP and ACT domains in the OSC GNs. The colour scale bar shows copy number values.

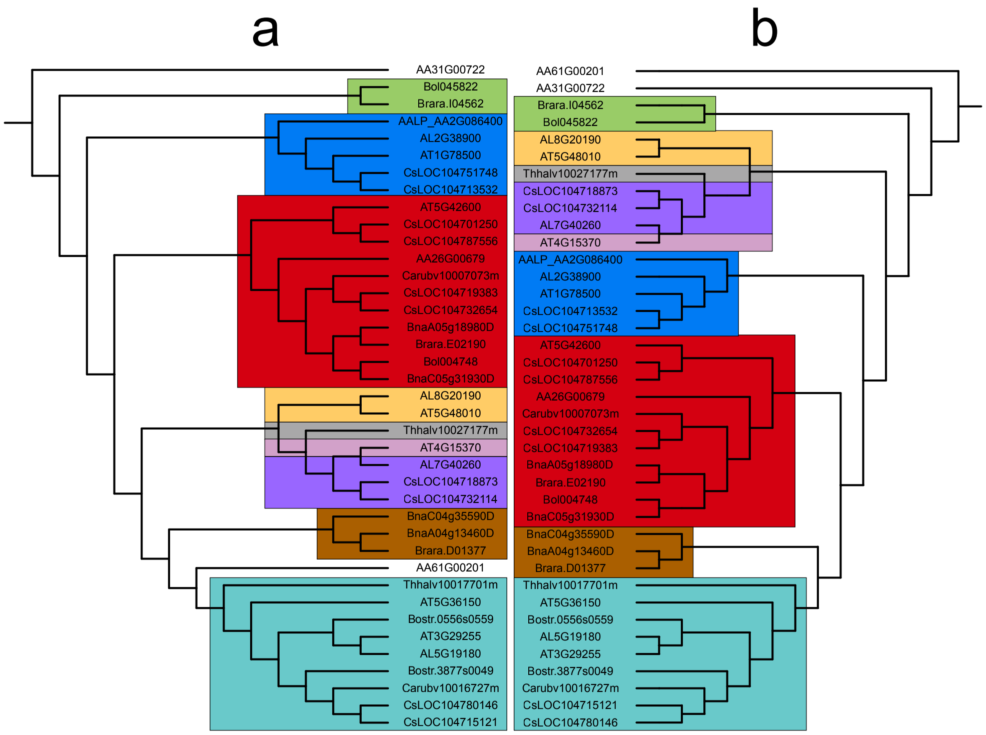

Fig. S2. Topology comparison of clade II OSC trees generated by **a)** RAxML and **b)** FastTree. Similar clades have been highlighted with the same color. The comparison highlights the differences concern deep ancestral splits in the OSC phylogeny that are difficult to resolve; as can be derived from comparison with Figure S6a, the same general conclusions regarding multiple parallel origins of triterpene biosynthetic loci would be reached when using the RAxML tree for ancestral state reconstruction.

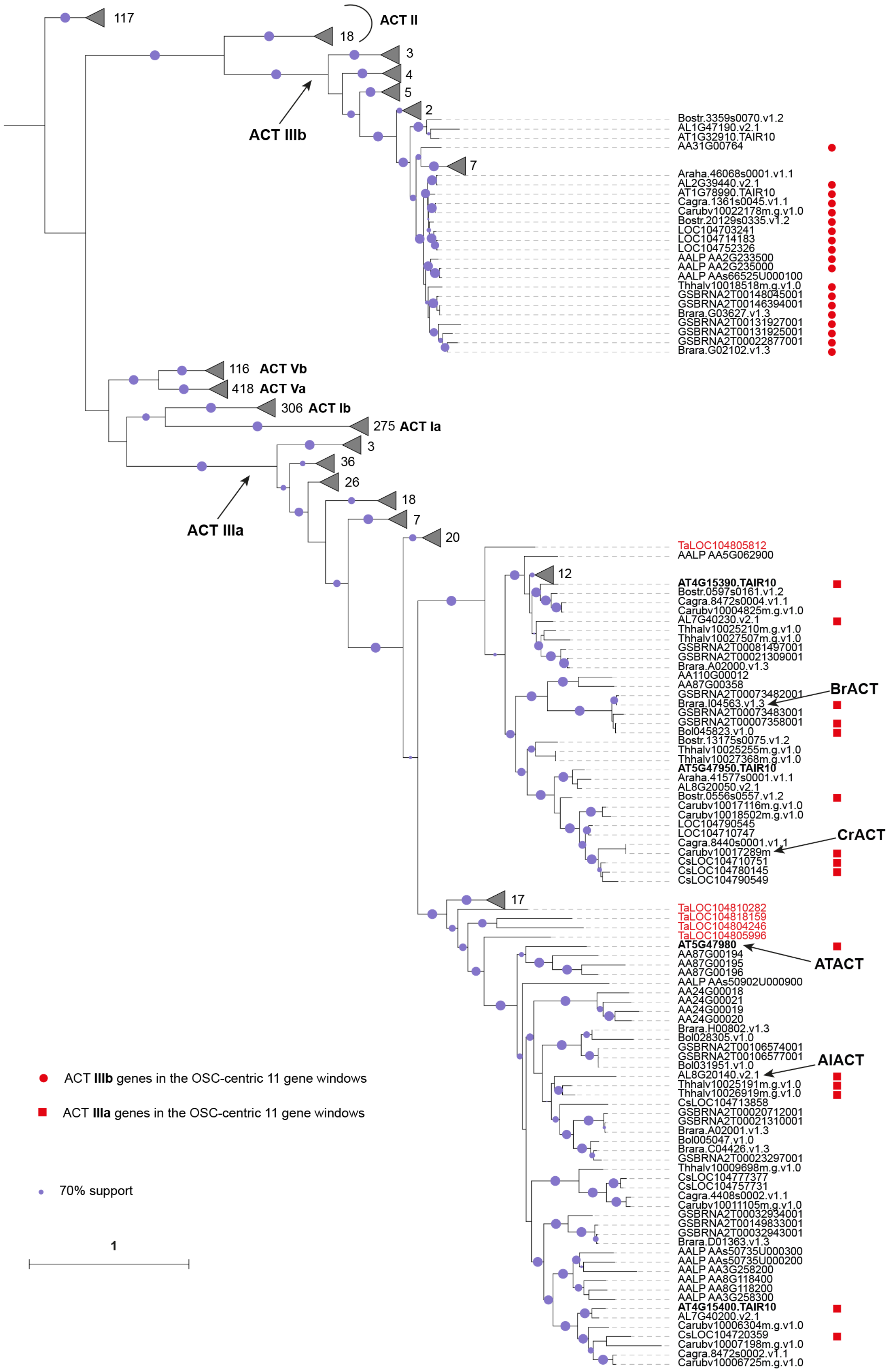
117
18
ACT II
3
4
5
2
7
ACT IIIb
Bostr.3359s0070.v1.2
AL1G47190.v2.1
AT1G32910.TAIR10
AA31G00764
Araha.46068s0001.v1.1
AL2G39440.v2.1
AT1G78990.TAIR10
Cagra.1361s0045.v1.1
Carubv10022178m.g.v1.0
Bostr.20129s0335.v1.2
LOC104703241
LOC104714183
LOC104752326
AALP AA2G233500
AALP AA2G235000
AALP AAs66525U000100
Thhalv10018518m.g.v1.0
GSBRNA2T00148045001
GSBRNA2T00146394001
Brara.G03627.v1.3
GSBRNA2T00131927001
GSBRNA2T00131925001
GSBRNA2T00022877001
Brara.G02102.v1.3
116 ACT Vb
418 ACT Va
306 ACT Ib
275 ACT Ia
3
36
26
18
7
20
ACT IIIa
12
17
TaLOC104805812
AALP AA5G062900
AT4G15390.TAIR10
Bostr.0597s0161.v1.2
Cagra.8472s0004.v1.1
Carubv10004825m.g.v1.0
AL7G40230.v2.1
Thhalv10025210m.g.v1.0
Thhalv10027507m.g.v1.0
GSBRNA2T00081497001
GSBRNA2T00021309001
Brara.A02000.v1.3
AA110G00012
AA87G00358
GSBRNA2T00073482001
Brara.I04563.v1.3
GSBRNA2T00073483001
GSBRNA2T00007358001
Bol045823.v1.0
Bostr.13175s0075.v1.2
Thhalv10025255m.g.v1.0
Thhalv10027368m.g.v1.0
AT5G47950.TAIR10
Araha.41577s0001.v1.1
AL8G20050.v2.1
Bostr.0556s0557.v1.2
Carubv10017116m.g.v1.0
Carubv10018502m.g.v1.0
LOC104790545
LOC104710747
Cagra.8440s0001.v1.1
Carubv10017289m
CsLOC104710751
CsLOC104780145
CsLOC104790549
BrACT
CrACT
TaLOC104810282
TaLOC104818159
TaLOC104804246
TaLOC104805996
AT5G47980
AA87G00194
AA87G00195
AA87G00196
AALP AAs50902U000900
AA24G00018
AA24G00021
AA24G00019
AA24G00020
Brara.H00802.v1.3
Bol028305.v1.0
GSBRNA2T00106574001
GSBRNA2T00106577001
Bol031951.v1.0
AL8G20140.v2.1
Thhalv10025191m.g.v1.0
Thhalv10026919m.g.v1.0
CsLOC104713858
GSBRNA2T00020712001
GSBRNA2T00021310001
Brara.A02001.v1.3
Bol005047.v1.0
Brara.C04426.v1.3
GSBRNA2T00023297001
Thhalv10009698m.g.v1.0
CsLOC104777377
CsLOC104757731
Cagra.4408s0002.v1.1
Carubv10011105m.g.v1.0
GSBRNA2T00032934001
GSBRNA2T00149833001
GSBRNA2T00032943001
Brara.D01363.v1.3
AALP AAs50735U000300
AALP AAs50735U000200
AALP AA3G258200
AALP AA8G118400
AALP AA8G118200
AALP AA3G258300
AT4G15400.TAIR10
AL7G40200.v2.1
Carubv10006304m.g.v1.0
CsLOC104720359
Carubv10007198m.g.v1.0
Cagra.8472s0002.v1.1
Carubv10006725m.g.v1.0
ATACT
AlACT
ACT IIIb genes in the OSC-centric 11 gene windows
ACT IIIa genes in the OSC-centric 11 gene windows
70% support
1

Fig. S3. Phylogeny of ACTs that are associated with clade I and clade II OSCs.

The maximum-likelihood tree was generated from an alignment of 1519 ACT protein sequences from seventeen genomes (see **Table S1**), with clades Ia/b, II and Va/b collapsed. The number of collapsed leaves is indicated. The cyan dot on branches denotes bootstrap support > 70% in 1000 replicates. The bar denotes amino acid substitutions per site. Well-annotated *A. thaliana* ACTs were used as marker genes and are highlighted in bold. ACTs associated with OSC-centric GNs are expanded. Red squares denote ACT IIIa proteins encoded by genes within clade II OSC GNs. Red circles indicate the ACT IIIb proteins encoded by genes within clade I OSC GNs. The previous characterized *A. thaliana* ACT (AtACT) and three characterized ACT in this study (AlACT from *A. lyrata*; CrACT from *C. rubella*; BrACT from *B. rapa*) are indicated by arrows. The ACT/OSC associations are limited to the Brassicaceae species. Genes from outgroup *Tarenaya hassleriana* are highlighted in red.

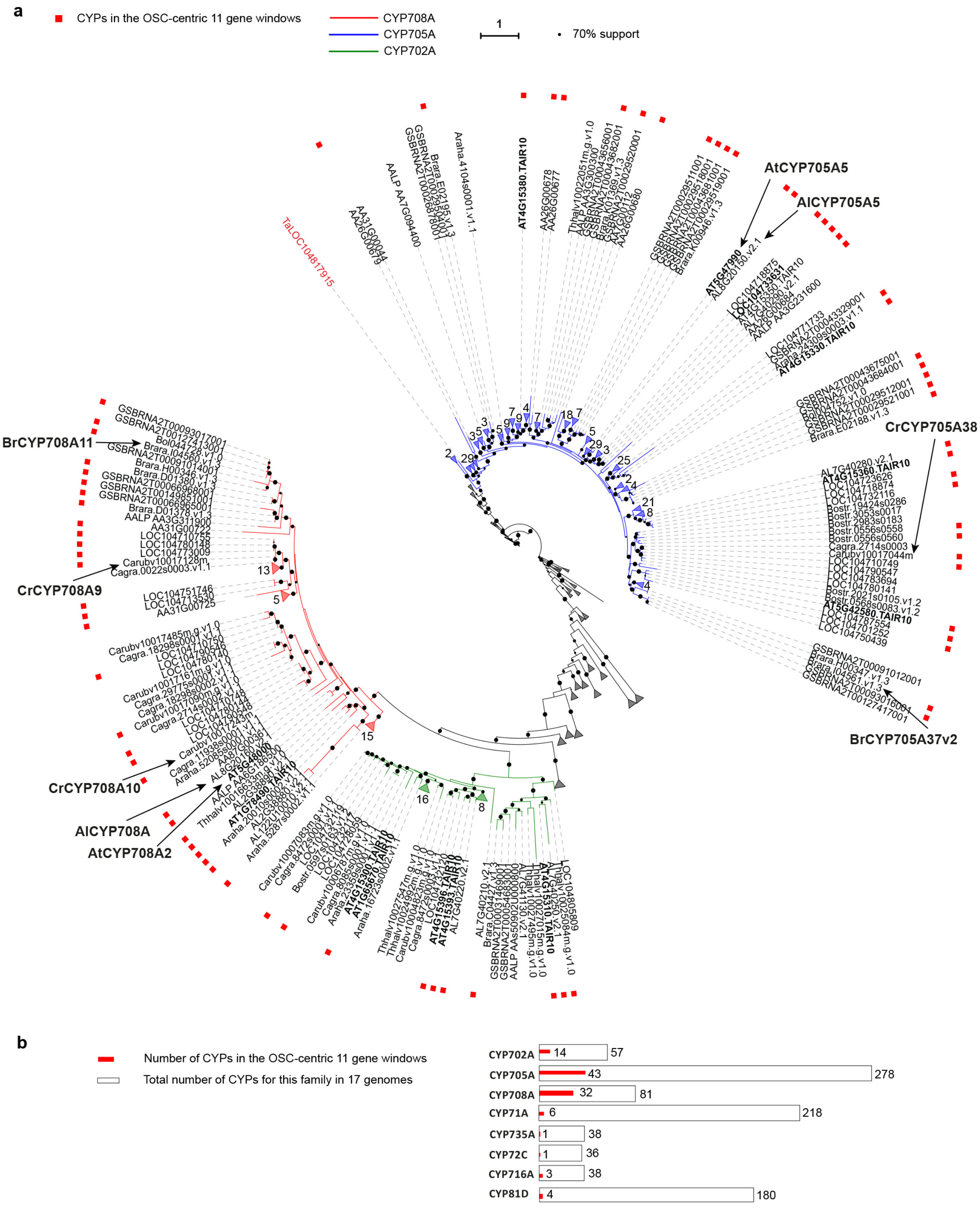
a
CYPs in the OSC-centric 11 gene windows
CYP708A
CYP705A
CYP702A
1
70% support
AtCYP705A5
AlCYP705A5
CrCYP705A38
BrCYP705A37v2
BrCYP708A11
CrCYP708A9
CrCYP708A10
AlCYP708A
AtCYP708A2
b
Number of CYPs in the OSC-centric 11 gene windows
Total number of CYPs for this family in 17 genomes
CYP702A 14 57
CYP705A 43 278
CYP708A 32 81
CYP71A 6 218
CYP735A 1 38
CYP72C 1 36
CYP716A 3 38
CYP81D 4 180

Fig. S4. Phylogeny of CYPs that are associated with clade II OSCs.

a, The maximum-likelihood tree was generated from an alignment of 5056 CYP protein sequences from seventeen genomes (see **Table S1**). Black dots on branches denote bootstrap support > 70% in 1000 replicates. The scale bar denotes amino acid substitutions per site. Well-annotated *A. thaliana* CYPs are highlighted in bold and are used as marker genes to assign the CYP families. The major CYP subfamilies (CYP705A, CYP708A and CYP702A) appeared in the clade II OSC GNs are shown. The CYPs from other families are collapsed. The number of collapsed leaves in CYP705A clade is indicated. The red squares denote the CYP proteins encoded by genes in the clade II OSC GNs. The previous characterized *A. thaliana* CYP (AtCYP705A5, AtCYP708A2) and seven cloned/characterized CYP in this study (AlCYP708A and AtCYP705A from *A. lyrata*; CrCYP708A9, CrCYP708A10 and CrCYP705A38 from *C. rubella*; BrCYP708A11 and BrCYP705A37v2 from *B. rapa*) are indicated by arrows. CYP708A and CYP702A are limited to Brassicaceae family. CYP705 is wildly spread in Brassicaceae, although one CYP705 like protein is found in outgroup *Tarenaya hassleriana* (highlighted in red). The CYP/OSC associations are limited to the Brassicaceae species. **b**, Eight CYP families were found within clade II OSC GNs. The total number of each identified CYP families is indicated by the empty boxes. The red bars denote numbers of CYPs present in OSC-centric 11 gene windows.

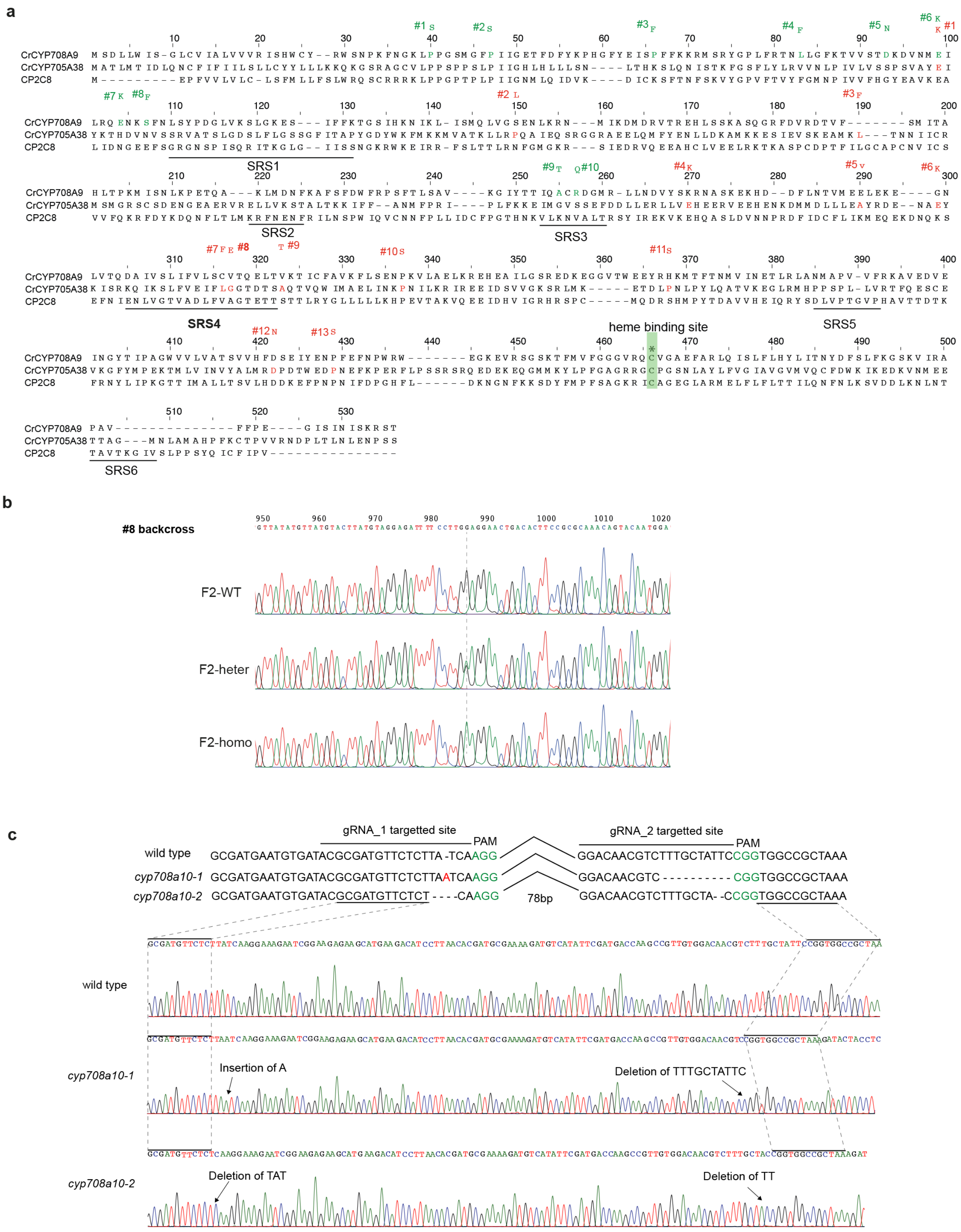

a
CrCYP708A9
CrCYP705A38
CP2C8
SRS1
SRS2
SRS3
SRS4
SRS5
SRS6
heme binding site
b
#8 backcross
F2-WT
F2-heter
F2-homo
c
gRNA_1 targetted site
gRNA_2 targetted site
PAM
wild type
cyp708a10-1
cyp708a10-2
78bp
Insertion of A
Deletion of TTTGCTATTC
Deletion of TAT
Deletion of TT

Fig. S5. Identification of *C. rubella* TILLING/CRISPR-Cas9 mutants for *CYP705A38* and *CYP708A10*.

a, Protein sequences were aligned with Muscle (Edgar, 2004) and the crystallized CYP2C8 (human metabolizing cytochrome P450 2C8) protein sequence was used for mapping conserved and divergent residues. SRSs (substrate recognition sites) and CYP heme binding site are indicated according to a previous study (Gotoh, 1992). The single nucleotide polymorphisms (SNPs) of TILLING mutants are shown above the mutation site. Eight independent mutant lines were identified for *CYP708A9.* No metabolic phenotype was observed in comparison to wild type. Thirteen independent mutant lines were identified for *CYP705A38. CYP705A38-*#8 was identified with a nonsynonymous mutation (G to E) in the SRS4 region. This is the only mutant line showed metabolic phenotype. **b**, *CYP705A38-*#8 was crossed with wild type. Individual plants from wild type, heterozygote and homozygote from F2 population were sequenced. Shown are sequencing result from representative samples. **c**, Sequence comparison of two independent *CYP708A10* CRISPR/Cas9 edited lines to the wild type. Two guide RNA targeted sites were designed before the protospacer adjacent motif (PAM) sites. Sequencing results of the two CRISPR edited lines are shown below. The edited positions (deletion or insertion) are indicated by arrows. The flanking non-edited regions are denoted with bars.

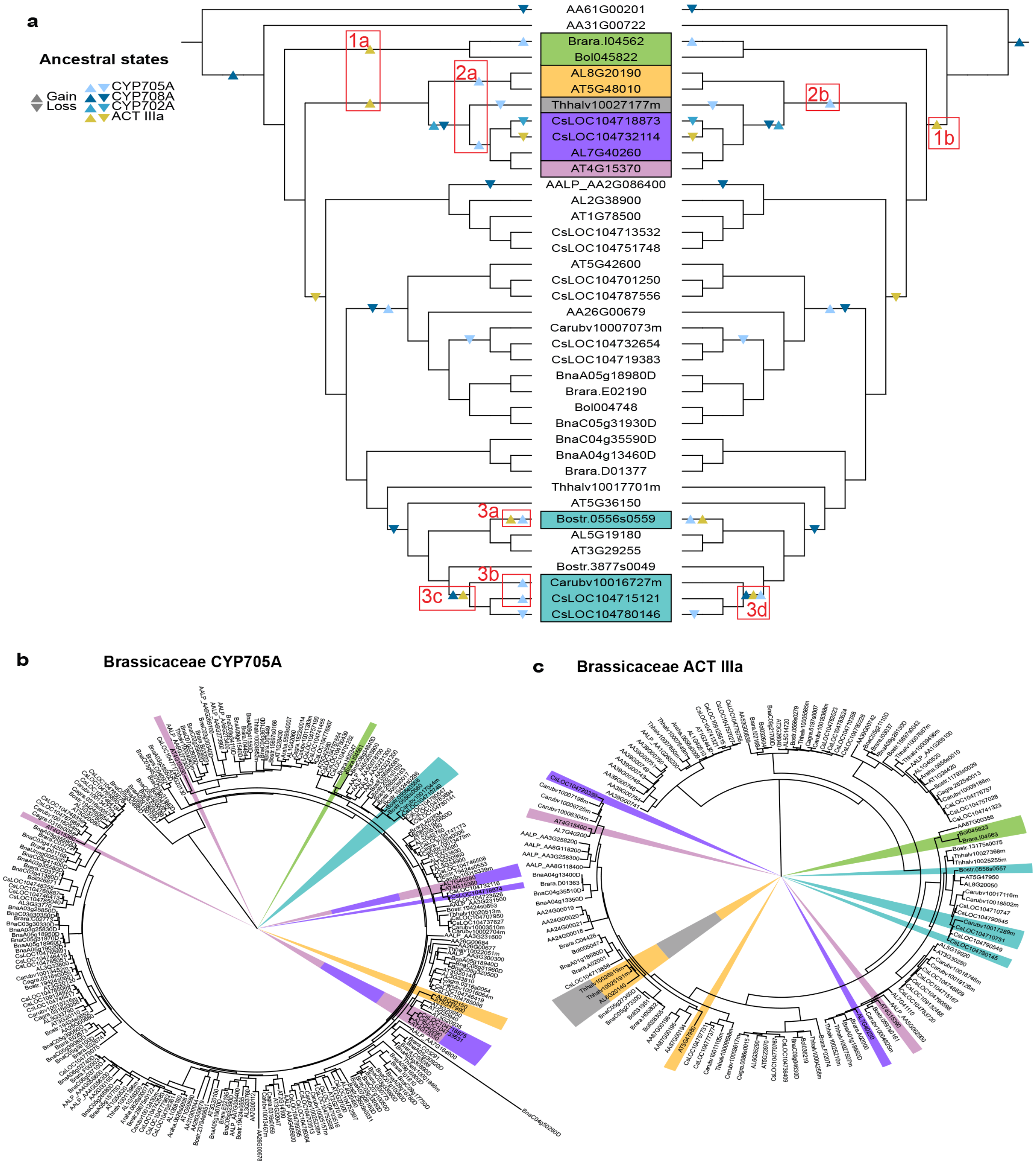
a
Ancestral states
Gain
Loss
CYP705A
CYP708A
CYP702A
ACT IIIa
AA61G00201
AA31G00722
Brara.I04562
Bol045822
AL8G20190
AT5G48010
Thhalv10027177m
CsLOC104718873
CsLOC104732114
AL7G40260
AT4G15370
AALP_AA2G086400
AL2G38900
AT1G78500
CsLOC104713532
CsLOC104751748
AT5G42600
CsLOC104701250
CsLOC104787556
AA26G00679
Carubv10007073m
CsLOC104732654
CsLOC104719383
BnaA05g18980D
Brara.E02190
Bol004748
BnaC05g31930D
BnaC04g35590D
BnaA04g13460D
Brara.D01377
Thhalv10017701m
AT5G36150
Bostr.0556s0559
AL5G19180
AT3G29255
Bostr.3877s0049
Carubv10016727m
CsLOC104715121
CsLOC104780146
1a
1b
2a
2b
3a
3b
3c
3d
b
Brassicaceae CYP705A
c
Brassicaceae ACT IIIa

Fig. S6. Ancestral states reconstruction of CYP and ACT subfamilies in clade II OSC GNs. Phylogenetic tree of Brassicaceae OSC enzymes with ancestral state changes (recruitment or loss of CYP702A, CYP705A, CYP708A and ACT IIIa into their surrounding genomic neighborhoods) reconstructed through maximum parsimony. **a** (Left), Ancestral states changes considering equivocal states. **a** (Right), Ancestral states changes when favouring early enzyme recruitment (all equivocal states = 1). The main differences between the trees are highlighted in red: the events 1a, 2a and 3b show parallel enzyme recruitment, while 1b, 2b and 3d show early recruitment and common ancestor neighborhoods. The event 3a appears in both trees, showing enzyme recruitment parallel to 3b/c/d. Genomic neighborhoods descending from these events are highlighted and all CYP705A and ACT IIIa genes in these clusters are equally highlighted in ***b*** & ***c***. **b**, Phylogenetic tree of Brassicaceae CYP705A enzymes. Highlighted leaves correspond to those CYP705A genes located in the GNs of OSCs shown in the phylogeny in panel ***a***. **c**, Phylogenetic tree of Brassicaceae ACT IIIa enzymes. Highlighted leaves correspond to those ACT IIIa genes located in the genomic neighborhoods of OSCs present in the phylogeny in panel ***a***.

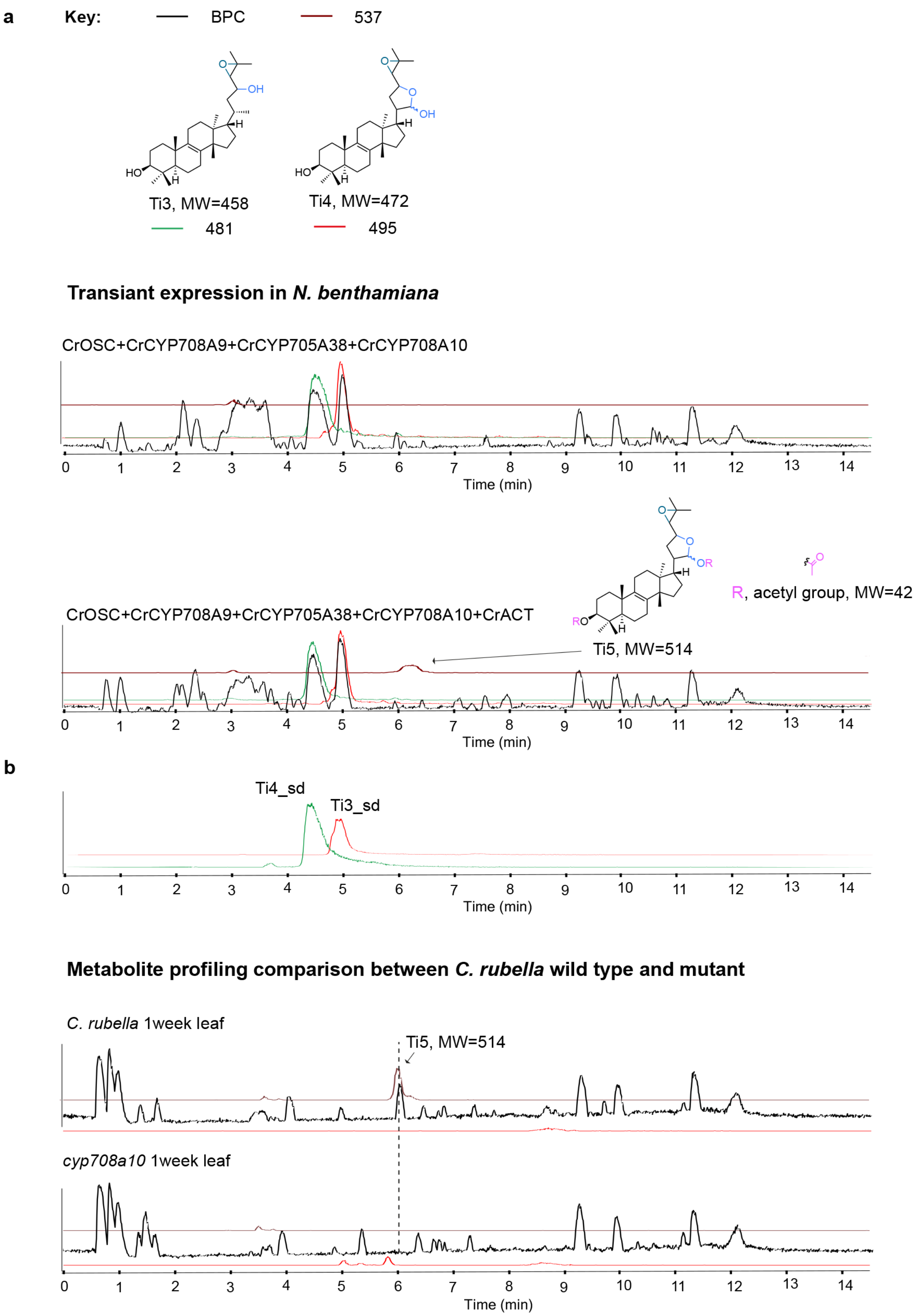
a
Key:
BPC
537
Ti3, MW=458
481
Ti4, MW=472
495
Transiant expression in N. benthamiana
CrOSC+CrCYP708A9+CrCYP705A38+CrCYP708A10
Time (min)
CrOSC+CrCYP708A9+CrCYP705A38+CrCYP708A10+CrACT
R, acetyl group, MW=42
Ti5, MW=514
Time (min)
b
Ti4_sd
Ti3_sd
Time (min)
Metabolite profiling comparison between C. rubella wild type and mutant
C. rubella 1week leaf
Ti5, MW=514
cyp708a10 1week leaf
Time (min)

Fig. S7. Functional analysis of the ACT from the *C. rubella* BGC.

a, LC-MS-IT-TOF analysis of *N. benthamiana* leaf extracts expression the *C. rubella* tirucallol BGC genes with or without ACT. Shown are the base peak chromatogram (BPC) and selected parent ion chromatograms (in different colours, see key). Purified **Ti3** (parent ion 458 + adduct ion 23 = 481) and **Ti4** (parent ion 472 + adduct ion 23 = 495) were used as control for running the LC-MS analysis. Targeted ion search of adding acetyl group (MW = 42) onto **Ti4** was performed. The **Ti5** peak (parent ion 504 + adduct ion 23 = 537) was only observed when ACT was present. **b**, LC-MS-IT-TOF analysis of the leaf extracts from *C. rubella* wild type and *cyp708a10* mutants. Chromatograms are shown in different colours (see key in panel ***a***). Peaks for **Ti5** are indicated by arrow.

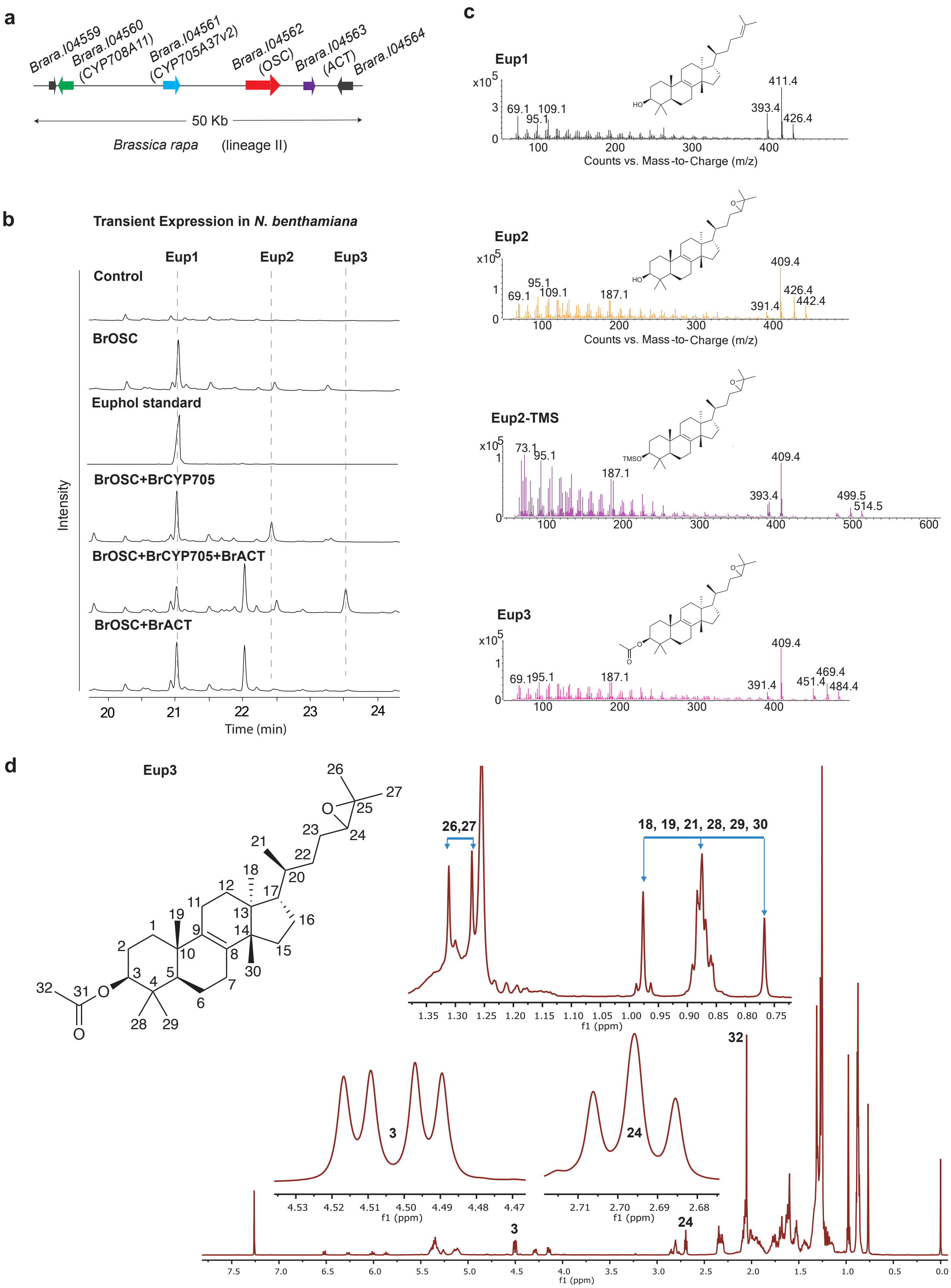
a
Brara.I04559
Brara.I04560 (CYP708A11)
Brara.I04561 (CYP705A37v2)
Brara.I04562 (OSC)
Brara.I04563 (ACT)
Brara.I04564
50 Kb
Brassica rapa (lineage II)
b
Transient Expression in N. benthamiana
Eup1
Eup2
Eup3
Control
BrOSC
Euphol standard
BrOSC+BrCYP705
BrOSC+BrCYP705+BrACT
BrOSC+BrACT
Intensity
Time (min)
c
Eup1
Eup2
Eup2-TMS
Eup3
Counts vs. Mass-to-Charge (m/z)
d
Eup3
f1 (ppm)
26,27
18, 19, 21, 28, 29, 30
3
24
32

Fig. S8. Functional characterization of *B. rapa* euphol BGC.

a, Structure of the *B. rapa* euphol BGC. **b**, GC-MS of total ion chromatogram of *N. benthamiana* leaf extract expressing euphol BGC genes. Eup**1-3** are used to label the new products when a new enzyme was introduced. The euphol standard (order from Avachem Scientific; CAS: 514-47-6) was used to characterize the Eup1. Note that ACT alone could modify euphol. **c**, ion spectra of Eup1-3 peaks and the underlying chemical structures. The Eup2 was analysed with or without derivatization treatment. **d**, ^{1}H spectra of Eup3, and the carbon numbering scheme of the proposed structure **eupha-8-en-24,25-epoxy-3β-yl acetate (600 MHz, $CDCl_3$ [referenced to TMS])**. Attempts to isolate the product of the transiently co-expressed *B.rapa* euphol synthase, CYP705A, and ACT for full NMR assignment failed. However, a ^{1}H spectra of partially purified fractions containing the product recorded during the purification process is consistent with a mixture containing a compound with the proposed structure of **eupha-8-en-24,25-epoxy-3β-yl acetate**. A doublet of doublets (1H, dd, *J*=11.9, 4.6) characteristic of the H-3α of euphane tetracycles was observed at the downfield position of δ 4.50 ppm. This is consistent with acetylation of the germinal hydroxyl group. Furthermore, a singlet δ 2.05 ppm (3H, s) consistent with the H_3-32 of the acetate group was also observed. These resonances match the literature for the previously characterised euphol acetate (Leong & Harrison, 1999). A triplet (1H, *J*=6.2) was observed at δ 2.70 ppm and is consistent with H-24 following epoxidation of the C24-25 alkene of euphol. Furthermore, two singlets (3H, s) representing the C26 and C27 methyl groups were observed to be shifted upfield at δ 1.31 and δ 1.27 relative to that observed in the spectra of euphol acetate (δ 1.69, δ 1.61). This is also consistent with epoxidation of the C24-25 alkene of euphol. Indeed, these resonances match those assigned to the equivalent positions in the previously reported constitutionally identical stereoisomer **lanost-8-en-24,25-epoxy-3β-yl acetate** (Nishitoba *et al.*, 1988).

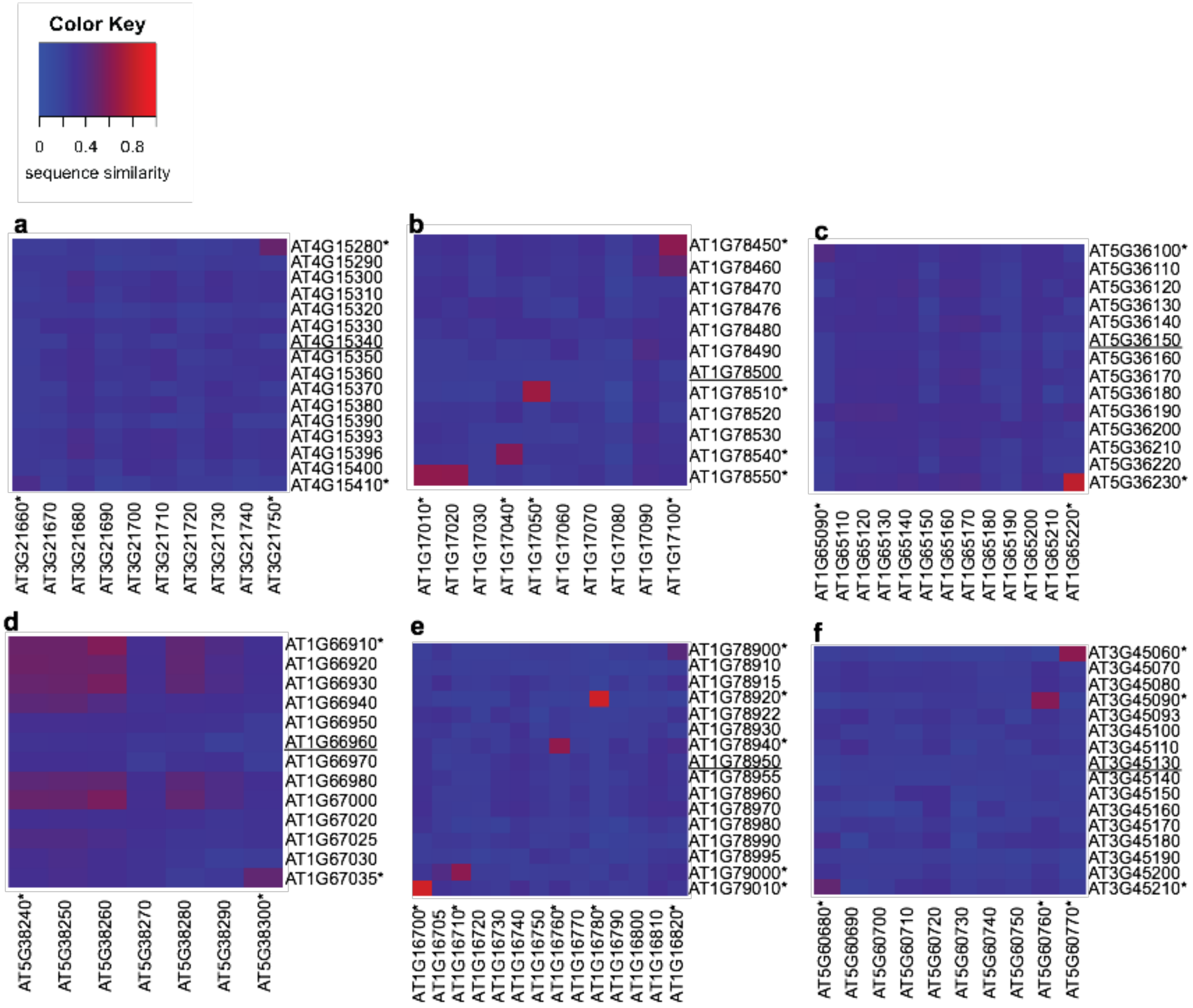

Fig. S9 Amino-acid sequence similarity of proteins encoded by *A. thaliana* OSC GNs located within WGD-derived syntenic block and their respective sister loci.

Heatmaps displaying the amino-acid sequence similarity matrix of proteins encoded by the *A. thaliana* OSC GNs located within WGD-derived syntenic blocks and their respective sister loci. OSC GNs are displayed vertically, and sister regions horizontally. Genes that encode OSCs are underlined. Anchor genes are marked with an asterisk. **a,** The arabidiol/baruol BGC within the *AT4G15340* GN is flanked by the anchor genes *AT4G15280* and *AT4G15410*, which define the sister region, flanked by the anchor genes *AT3G21660* and *AT3G21750*. **b,** The *AT1G87500* clade II OSC GN is flanked by the anchor genes *AT1G78450* and *AT1G78550*, which define its sister region, flanked by the anchor genes *AT1G16700* and *AT1G16820*. **c,** The tirucalla BGC

within the *AT5G36150* GN is flanked by the anchor genes *AT5G36100* and *AT5G36230*, which define its sister region, flanked by the anchor genes *AT1G65090* and *AT1G65220*. **d,** The *AT1G66960* clade I OSC GN is flanked by the anchor genes *AT1G66910* and *AT1G67035*, which define its sister region, flanked by the anchor genes *AT5G38240* and *AT5G38300*. **e,** The *AT1G78950* clade I OSC GN is flanked by the anchor genes *AT1G78900* and *AT1G79010*, which define its sister region, flanked by the anchor genes *AT1G16700* and *AT1G16820*. **f,** The *AT3G45130* sterol OSC GN is flanked by the anchor genes *AT3G45060* and *AT3G45210*, which define its sister region, flanked by the anchor genes *AT5G60680* and *AT5G60770*.

2. Supplementary Tables

Supplemental Tables S3, 5-8 are uploaded as Auxiliary Supplementary Materials.

Table S3. Revised sequence for functional analysis.

Table S5. 126 OSC-centric GNs identified in this study.

Table S6. GN associations with different regression analysis.

Table S7. Fisher's exact test on individual genomes.

Table S8. BIG-SCAPE index values and average amino acid identity of GN pairs.

Table S1. 163 Brassicaceae OSCs identified in this study.

Thirteen Brassicaceae genomes (one early diverged species, seven lineage I species, five lineage II species) and four closely related outgroup genomes were included in this analysis. The taxonomic rank of species is taken from (Beilstein *et al.*, 2010). The star denotes polyploid species. The numbers of identified OSCs using pHMMER, BlastP and phylogenetic analysis are listed, with the Brassicaceae ones labelled in red (in sum, 163). The genome source and assembly version for each genome are displayed in the last two columns, respectively.

Species	pHMMER	BlastP	Phylogeny	Source	Assembly version	Order/Family/Lineage
Arabidopsis thaliana	15	14	14	phytozome12	TAIR10	Brassicales, Brassicaceae, lineage I
Arabidopsis lyrata	19	17	18	phytozome12	v2.1	Brassicales, Brassicaceae, lineage I
Arabidopsis halleri	12	12	12	phytozome12	v1.1	Brassicales, Brassicaceae, lineage I
Camelina sativa *	27	24	24	NCBI	GCF_000633955.1	Brassicales, Brassicaceae, lineage I
Capsella grandiflora	8	7	7	phytozome12	v1.1	Brassicales, Brassicaceae, lineage I
Capsella rubella	8	7	7	phytozome12	v1.0	Brassicales, Brassicaceae, lineage I
Boechera stricta	7	7	7	phytozome12	v1.2	Brassicales, Brassicaceae, lineage I
Brassica oleracea	10	10	10	phytozome12	v1.0	Brassicales, Brassicaceae, lineage II
Brassica rapa	10	10	10	phytozome12	v1.3	Brassicales, Brassicaceae, lineage II
Brassica napus *	25	25	25	NCBI	GCA_000751015.1	Brassicales, Brassicaceae, lineage II
Thellungiella halophila	9	9	9	phytozome12	v1.0	Brassicales, Brassicaceae, lineage II
Arabis alpina	11	10	11	NCBI	GCA_000733195.1	Brassicales, Brassicaceae, lineage II
Aethionema arabicum	10	9	9	CoGE	v2.5, id32040	Brassicales, Brassicaceae, early digerging
Tarenaya hassleriana	14	14	14	NCBI	GCF_000463585.1	Brassicales, Cleomaceae
Carica papaya	14	12	13	phytozome12	ASGPBv0.4	Brassicales, Caricaceae
Gossypium raimondii	17	16	16	phytozome12	v2.1	Malvales, Malvaceae
Theobroma cacao	11	10	10	phytozome12	v1.1	Malvales, Malvaceae

Table S2. Primers used in this study.

Gene_ID	sequence	Note
at5g47960	CACTGCTGGTCAAGAACGGTATC	qRT-Fwd, span exon
at5g47960	ACCATCTCGCTACATGATCAAACG	qRT-Rev, span exon
at5g47980	ATGGCGATGTGCCAGAAATGCC	qRT-Fwd
at5g47980	TGAGTCATCATTGTGGCCCTTGG	qRT-Rev
at5g47990	TGTTGTTGGAAGCTTATGGAGACG	qRT-Fwd, span exon
at5g47990	TGCCTGCACTGAACAGATCCAC	qRT-Rev, span exon
at5g48000	AAAGCTCTCGCAGAGTTGAAGAG	qRT-Fwd, span exon
at5g48000	CCCAGCTAACTCCAGCTCCTTTAC	qRT-Rev, span exon
at5g48010	TTACCGGTTCATCGTGCTGCTC	qRT-Fwd, span exon
at5g48010	CGTTCCCATTATTTCCTGCTGTGG	qRT-Rev, span exon
at5g48020	TCTGAGCCTGTTGTACCGGAAG	qRT-Fwd, span exon
at5g48020	GCTGCCATTTCAGCAACAACCTC	qRT-Rev, span exon
at3G18780	CTTGCACCAAGCAGCATGAA	reference gene
at3G18780	CCGATCCAGACACTGTACTTCCTT	reference gene
Al8G20190	TTCGAGAGCTTGCCATCAGAGC	qRT-Fwd
Al8G20190	GCTATGCCTCCATTATCACTCTGC	qRT-Rev
AL8G20150	AAGTCCTTGTTCGTGGATCTGTTC	qRT-Fwd, span exon
AL8G20150	CTGTATAGTGTTCGCCGAGGATTC	qRT-Rev, span exon
AL8G20160	GCCAGGGTAGCTTCAATGCTAAGG	qRT-Fwd, span exon
AL8G20160	GGGTCAGGTGTGCCATTATCAAGC	qRT-Rev, span exon
Al8G20140	ACACGTGTGGAAGCCATCACATC	qRT-Fwd, span exon
Al8G20140	ACCAATTGCATCTCGGGAATCGC	qRT-Rev, span exon
AL1G25080	CATTTCACTCCTCTGGCTAAGCG	reference gene
AL1G25080	CATGCTGATACTCTGGCTGTGAAC	reference gene
Carubv10016727m	TGGCGATTTCCCACAAGAGGAC	qRT-Fwd, span exon
Carubv10016727m	AGTGAAGCAATCGTAGAGCCTTTG	qRT-Rev, span exon
Carubv10017289m	ACCAAGGACGGACAAGGAGTTG	qRT-Fwd
Carubv10017289m	TGTTCTGCTCGAACCGTACCATC	qRT-Rev
Carubv10017243m	GGCACGCAAATCTGCGATGAATG	qRT-Fwd
Carubv10017243m	TTGTCCACAACGGCTTGGTCATC	qRT-Rev
Carubv10017044m	CAAGCGACGGTTAAGGAAGGAC	qRT-Fwd
Carubv10017044m	TGTTCTAACCAAGAGCGGCGATG	qRT-Rev
Carubv10017128m	ACTCAGGAGCTCACCGTTAAGACC	qRT-Fwd, span exon
Carubv10017128m	AGGATTGCCTCGTGCTCTCTCTTC	qRT-Rev, span exon
Carubv10016708m	TCGCTCGAGCCGACAATTCTTC	qRT-Fwd, span exon
Carubv10016708m	AGCTTCTGAATGTTTGGACCTGAC	qRT-Rev, span exon
Carubv10018494m	ATGGCATGAGGCGCAAGGTATG	qRT-Fwd
Carubv10018494m	TTGATCCCACAGGGCTGGTTAC	qRT-Rev
Carubv10013961m	ACAGTGTCTGGATCGGTGGTTC	reference gene
Carubv10013961m	CCTTGGAGATCCACATCTGCTG	reference gene

Gene_ID	sequence	Note
Carubv10017044m	CAAGCGACGGTTAAGGAAGGAC	qRT-Fwd
Carubv10017044m	TGTTCTAACCAAGAGCGGCGATG	qRT-Rev
Carubv10017128m	ACTCAGGAGCTCACCGTTAAGACC	qRT-Fwd, span exon
Carubv10017128m	AGGATTGCCTCGTGCTCTCTCTTC	qRT-Rev, span exon
Carubv10016708m	TCGCTCGAGCCGACAATTCTTC	qRT-Fwd, span exon
Carubv10016708m	AGCTTCTGAATGTTTGGACCTGAC	qRT-Rev, span exon
Carubv10018494m	ATGGCATGAGGCGCAAGGTATG	qRT-Fwd
Carubv10018494m	TTGATCCCACAGGGCTGGTTAC	qRT-Rev
Carubv10013961m	ACAGTGTCTGGATCGGTGGTTC	reference gene
Carubv10013961m	CCTTGGAGATCCACATCTGCTG	reference gene
Carubv10016727m	ATGTGGAGGTTAAAGATCGGAG	gene cloning
Carubv10016727m	TTAGTGAAGCAATCGTAGAGC	gene cloning
Carubv10017289m	ATGGTTACACTCAAGGTTGAAAC	gene cloning
Carubv10017289m	TTACATCTTGGATAAAAGATAACG	gene cloning
Carubv10017243m	ATGAACATCATTTGGAACATTTC	gene cloning
Carubv10017243m	TCAACTAGTGGGAGTAGCATG	gene cloning
Carubv10017044m	ATGGCGACACTAATGACCATTG	gene cloning
Carubv10017044m	TCATGAACTCGGATTCTCAAG	gene cloning
Carubv10017128m	ATGAGTGATTTGTTGTGGATC	gene cloning
Carubv10017128m	TCATGTGGAACGTTTGGAGATG	gene cloning
AL8G20140	ATGGAAGCAAAGCTAGAGGTTG	gene cloning
AL8G20140	TTAGATCAAAACTGGAGGATTAAG	gene cloning
AL8G20150	ATGGCATCAATGATCACCGTTG	gene cloning
AL8G20150	TTAAGTGTTTAGGGTTCGAGG	gene cloning
AL8G20160	ATGAGCTTCGTCTGGTCCGCTG	gene cloning
AL8G20160	CTAGAGTGACTGGGAAATCTTG	gene cloning
Al8G20190	ATGTGGAGGCTGAGACTTGGAC	gene cloning
Al8G20190	TTAAGGGAGAAGCCGTCGCAG	gene cloning
L2F	GTGGTGTAAACAAATTGACGC	CRISPR sequencing
L2R	GGATAAACCTTTTCACGCCC	CRISPR sequencing
CRISPR_Rev	tgtggtctcaAGCG AAAAAAAGCACCGACTC	CRISPR reverse primer targetting gRNA backbone
Carubv10017243m	tgtggtctca ATTG TACGCGATGTTCTCTTATCA GTTTAAGAGCTATGCTGGAA	CrCYP708A10_sgRNA1_Fwd
Carubv10017243m	tgtggtctca ATT GGACAACGTCTTTGCTATTC GTTTAAGAGCTATGCTGGAA	CrCYP708A10_sgRNA2_Fwd
Carubv10017044m	CTGATGATCTTTTTATCTTTCTCTTC	TILLING_F1
Carubv10017044m	CTGATGATCTTTTTATCTTTCTCTTC	TILLING_R1
Carubv10017044m	CTGATGATCTTTTTATCTTTCTCTTC	TILLING_F3
Carubv10017044m	CTGATGATCTTTTTATCTTTCTCTTC	TILLING_R3

Table S4. NMR data.

a. ^{1}H δ information for **tirucallol (Ti1).**

The ^{1}H NMR result is consistent with the literature (Morlacchi *et al.*, 2009).

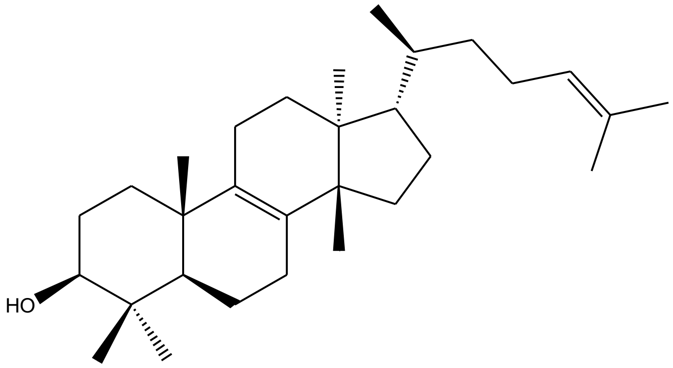

Tirucallol ^{1}H NMR ($CDCl_3$, 400 MHz) [referenced to TMS] *δ*. 5.10 (1H, tq, *J*=7.1, 1.4), 3.24 (1H, dd, *J*=11.6, 4.6), 2.13-0.82 (23H, m), 1.68 (3H, s), 1.60 (3H, s), 1.00 (3H, s), 0.95 (3H, s), 0.92 (3H, d, *J*=6.3), 0.87 (3H, s), 0.80 (3H, s), 0.76 (3H, s)

b. ^{13}C & ^{1}H δ assignments for **tirucalla-8,24-diene-3β,23-diol (Ti2)**. **$CDCl_3$** [referenced to TMS]. Coupling constants are reported as observed and not corrected for second order effects. Assignments were made via a combination of ^{1}H, ^{13}C, DEPT-edited HSQC, HMBC and 2D NOESY experiments where signals overlap 1H δ is reported as the centre of the respective HSQC cross peak.

Carbon numbering scheme and selected COSY and HMBC

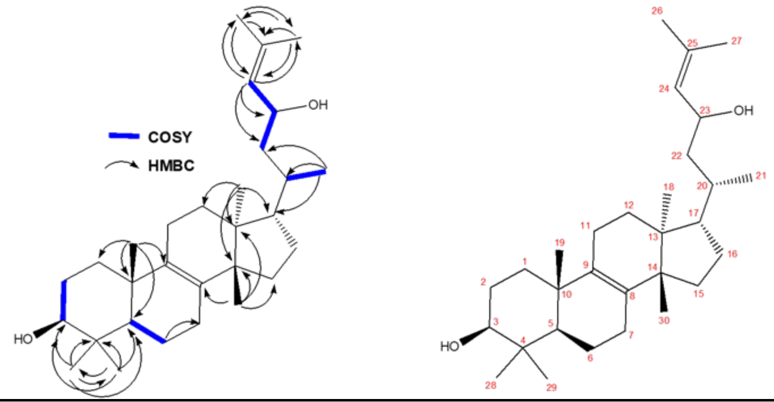

Carbon #	^{13}C δ	^{1}H δ	Carbon #	^{13}C δ	^{1}H δ
**25**	135.68	/	**12**	30.80	1.71 (1H, m)<br>1.68 (1H, m)
**9**	134.08	/	**15**	29.79	1.52 (1H, m)<br>1.20 (1H, m)
**8**	133.47	/	**16**	28.32	1.95 (1H, m)<br>1.28 (1H, m)
**24**	128.44	5.11 (1H, br d, *J*=9.0)	**28**	28.05	1.00 (3H, s)
**3**	78.99	3.24 (1H, dd, *J*=11.7, 4.4)	**2**	27.94	1.68 (1H, m)<br>1.60 (1H, m)
**23**	67.26	4.45 (1H, td, *J*=9.3, 4.0)	**7**	27.66	2.08 (1H, m)<br>1.92 (1H, m)
**5**	50.95	1.12(1H, m)	**26**	25.87	1.74 (3H, s)
**17**	50.69	1.50 (1H, m)	**30**	24.36	0.87 (3H, s)
**14**	50.01	/	**11**	21.44	2.05 (1H, m)<br>1.93 (1H, m)
**22**	44.53	1.57 (1H, m)<br>1.34 (1H, m)	**19**	20.15	0.95 (3H, s)
**13**	44.10	/	**21**	19.55	0.93 (3H, d, *J*=5.8)
**4**	38.94	/	**6**	18.93	1.68 (1H, m)<br>1.42 (1H, m)
**10**	37.27	/	**27**	18.32	1.71 (3H, s)
**1**	35.25	1.77 (1H, m)<br>1.20 (1H, m)	**29**	15.53	0.80 (3H, s)
**20**	33.97	1.33 (1H, m)	**18**	15.37	0.74 (3H, s)

c. ^{13}C & ^{1}H δ assignments for **tirucalla-24,25-epoxy-3β,23-diol** (**Ti3**). **CDCl₃** [referenced to TMS]. Coupling constants are reported as observed and not corrected for second order effects. Assignments were made via a combination of ^{1}H, ^{13}C, DEPT-edited HSQC, HMBC and 2D NOESY experiments. Where signals overlap ^{1}H δ is reported as the centre of the respective HSQC cross peak.

Carbon numbering scheme and selected COSY and HMBC

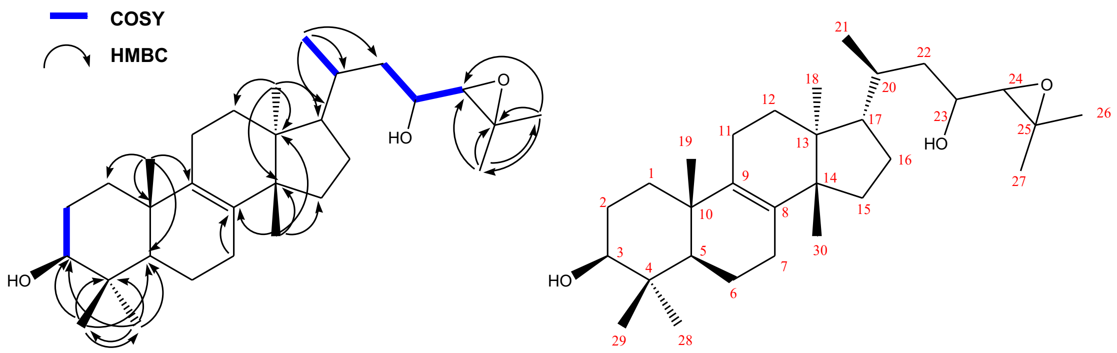

Carbon #	^{13}C δ	^{1}H δ	Carbon #	^{13}C δ	^{1}H δ
**9**	135.27	/	**12**	31.49	1.78 (2H, m)
**8**	134.06	/	**15**	30.66	1.65 (1H, m)<br>1.29 (1H, m)
**3**	78.55	3.51 (1H, m)	**16**	29.40	2.13 (1H, m)<br>1.46 (1H, m)
**23**	70.34	3.98 (1H, m)	**2**	29.33	1.95 (2H, m)
**24**	70.08	3.05 (1H, d, *J*= 8.3)	**29**	29.18	1.28 (3H, s)
**25**	59.15	/	**7**	28.51	2.14 (1H, m)<br>2.00 (1H, m)
**5**	51.98	1.29 (1H, m)	**26**	25.59	1.39 (3H, s)
**17**	51.45	1.73 (1H, m)	**30**	25.04	0.99 (3H, s)
**14**	50.75	/	**11**	22.25	2.12 (1H, m)<br>2.03 (1H, m)
**13**	44.94	/	**21**	21.55	1.20 (3H, d, *J*=5.0)
**22**	42.66	1.92 (1H, m)<br>1.72 (1H, m)	**19**	20.91	1.07 (3H, s)
**4**	40.06	/	**27**	20.65	1.41 (3H, s)
**10**	38.11	/	**6**	19.85	1.78 (1H, m)<br>1.49 (1H, m)
**1**	36.32	1.79 (1H, m)<br>1.28 (1H, m)	**28**	16.99	1.11 (3H, s)
**20**	35.08	1.73 (1H, m)	**18**	16.02	0.91 (3H, s)

d. ^{13}C & ^{1}H δ assignments for **tirucalla-24,25,21,23-diepoxy-3β,21-diol (Ti4)** (isolated as a C21 epimeric mixture). **$CDCl_3$ [referenced to TMS]**. Coupling constants are reported as observed and not corrected for second order effects. Assignments were made via a combination of ^{1}H, ^{13}C, DEPT-edited HSQC, HMBC and 2D NOESY experiments Where signals overlap 1H δ is reported as the centre of the respective HSQC cross peak.

Carbon numbering scheme and selected COSY and HMBC

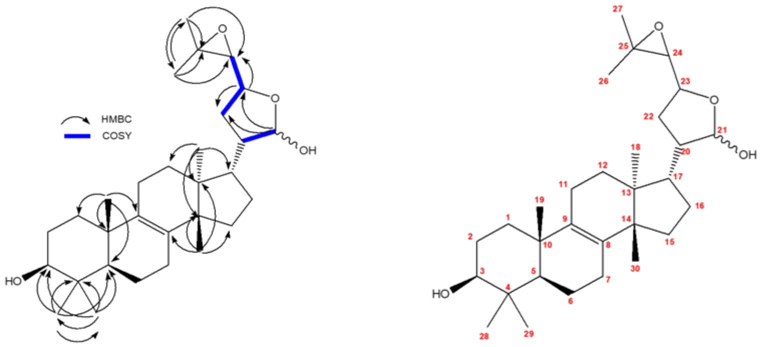

Carbon #	^{13}C δ		^{1}H δ	Carbon #	^{13}C δ		^{1}H δ
**9**	134.33	134.16	/	**1**	35.25	35.20	2H (1.78, m, 1.2, m)
**8**	133.32	133.22	/	**15**	30.25	30.12	2H (1.80, m, 1.46, m) (1.60, m, 1.27, m)
**21**	102.57	97.73	1H (5.32, d, *J*=2.6) (5.39, brs)	**12**	29.67	29.46	1.68 (2H, m)
**3**	78.97	78.93	3.23 (1H, dd, *J*=11.6, 4.6)	**28**	28.06		1.05 (3H, s)
**23**	78.39	77.11	1H (3.87, m) (3.92, m)	**7**	27.91		1.63 (2H, m)
**24**	67.75	65.27	1H (2.84, d, *J*=7.5) (2.71, d, *J*=7.5)	**2**	27.65		2H (2.11, m, 1.93, m)
**25**	57.92	57.20	/	**16**	27.20	26.98	2H (1.87, m, 1.37, m)
**5**	50.98	50.96	1.12 (1H, m)	**26**	25.01	24.92	3H (1.33, s, 1.32, s)
**20**	49.64	47.48	1H (2.24, m) (2.05, m)	**30**	24.54	24.38	3H (0.91, s, 0.89, s)
**14**	49.57	49.41	/	**11**	21.47	21.31	2H (2.04, m)
**13**	44.30	44.05	/	**19**	20.17		0.96 (3H, s)
**17**	47.73	43.29	1H (1.83, m) (2.07, m)	**27**	19.45	19.22	1.31 (3H, s)
**4**	38.94		/	**6**	18.91		2H (1.70, m, 1.42, m)
**10**	37.32		/	**18**	17.19	16.61	3H (0.79, s, 0.83, s)
**22**	35.38	31.80	2H (2.11, m, 1.37, m) (2.04, m, 1.71 m)	**29**	15.52		0.80 (3H, s)

Table S9. The relationship of OSC-centric GNs and WGD-derived ACK blocks.

OSCs located in an ACK block different from the other closely related OSCs are marked with an asterisk.

Arabidopsis thaliana		Other Brassicaceae species			Notes
OSC	ACK block	Closely related OSC	Species	ACK block	
AT1G66960	E	AL2G25240<br>AALP_AA3G344300*	*A. lyrata*<br>*A. alpina*	E<br>H	This suggests that AALP_AA3G344300 has been translocated from the E block to the H block, or that the corresponding OSC in the *A. thaliana/A. lyrata* common ancestor has been translocated
AT1G78500	E	AL2G38900<br>AALP_AA2G086400	*A. lyrata*<br>*A. alpina*	E	
AT1G78950, AT1G78955, AT1G78960, AT1G78970	E	AL2G39400<br>AALP_AA2G234300	*A. lyrata*<br>*A. alpina*	E	
AT2G07050	G	AL3G44070<br>Carubv10013036m	*A. lyrata*<br>*C. rubella*	G	According to Murat et. al, (2015) Carubv10013036m is in the pericentromeric region between G and H, but very close to the
AT3G29255	L	AL5G19180<br>Carubv10016727m (Tirucallol)	*A. lyrata*<br>*C. rubella*	L	
AT3G45130	M	AL5G23630<br>AALP_AA5G078800<br>AALP_AA5G037300*	*A. lyrata*<br>*A. alpina*	M<br>K-L	This suggests that AALP_AA5G078800 and AALP_AA5G037300 are the result of a duplication, after which the latter has been translocated from the M block to the K-L region.
AT4G15340, AT4G15370 (Arabidiol / Baruol)	T	AL7G40260	*A. lyrata*	T	
AT5G36150 (Tirucalla)	S	Brara.D01377*	*B. rapa*	I	
AT5G42600 (Marneral)	V	Brara.E02190*	*B. rapa*	F	
AT5G48010 (Thalianol)	W	AL8G20190	*A. lyrata*	W	According to Murat et. al, AL8G20190 is in the pericentromeric region between V and W, but very close to the latter.
-	-	Brara.I04562 (euphol)	*B. rapa*	I	No *A. thaliana* OSC is grouped to *B. rapa*'s euphol OSC in the phylogeny.

3. Notes S1

Genomic Neighbourhoods (GN) exploration and protein domain enrichment

We identified 163 oxidosqualene cyclase (OSC) genes, 5056 cytochrome P450 (CYP) genes and 1519 acyltransferase (ACT) genes throughout 13 Brassicaceae genomes. By querying all genomes for all genomic neighbourhoods around the 163 OSC genes, we identified a total of 126 unique OSC neighbourhoods (50 for clade II OSCs, 48 for clade I OSCs, and 28 for sterol clade OSCs) (Table S5). Within these GNs, we found 438 unique protein domains. The five most abundant domains were Pkinase_Tyr (PF07714), Pkinase (PF00069), P450, Transferase and LRRNT_2 (PF08263), with 100, 99, 96, 34 and 27 appearances, respectively. We identified notable differences between the genomic neighbourhoods of the three OSC subfamilies: 159 unique protein domains were identified around clade I OSCs, 201 for clade II OSCs, and 162 around sterol OSCs; however, only 30 domains were shared between clade I and clade II GNs, 31 domains between clade I and sterol, 31 between clade II and sterol, and only 8 domains appeared in all three. Clade I OSC GNs show the highest number of enriched domains (15 domains with $P<0.01$, conservative Fisher's exact test [see **Materials and Methods**]), followed by clade II and sterol GNs with 8 and 5 enriched domains respectively (Table S6). The enriched domains were mostly unique for one of the three groups, with only the Transferase domain (covering BAHD acyltransferases) being enriched in both clade I and clade II neighborhoods. Interestingly, the P450 and Transferase domains were enriched in clade II OSC genomic neighborhoods, and so were the CYP708A, CYP705A and ACT IIIa subfamilies, which also comprise the tailoring enzymes found in the previously reported thalianol pathway (Field & Osbourn, 2008).

The close phylogenetic relationships between the species in our study represents a problem for standard statistical procedures in part due to the non-independence of the samples (Graber, 2013). In order to reduce the problems that arise from this, we selected two additional methods that incorporate phylogenetic information into statistical procedures to complement our initial exploration of enriched domains: phylogenetic logistic regression (PLR), and phylogenetic generalized linear mixed models (PGLMM) [see **Materials and Methods**]. We used these methods to test in all OSC GNs whether the presence of a specific OSC subfamily was a strong predictor for the presence of other protein families in the same GN (this time disregarding

enrichment of these families in the GNs versus the rest of the genome, for which the Fisher's exact test was used). This is done by examining the distribution of protein families within the OSC GNs and measuring their phylogenetic predisposition within the OSC phylogeny, ensuring significant associations are not simply the result of shared ancestry. The PLR model shows that the presence of a clade II OSC (rather than a clade I or sterol-clade OSC) is a strong predictor for the presence of a CYP705A in the genomic region around the OSC (P=0.004); however, within the PGLMM model, the association is not significant (P=0.177). In contrast to the results of Fisher's exact test, clade II OSCs were not shown to be a significant predictor for any of the remaining potential tailoring enzyme subfamilies in the regression models. This might be because the sparse/irregular distribution of the gene families across the OSC phylogeny. Interestingly, the effect size in the association between clade II OSCs and ACT IIIa was the largest of all tests in both regression models (Odds Ratio(PLR)=3.34E08 and OR(PGLMM)=2.02E11). Large effect size in conjunction with non-significant P-values often indicate the need for methods with more statistical power and/or a larger sample size.

In conclusion, the results of these tests indicate the GNs of the three OSC clades have a distinct evolutionary history, protein domain content and biosynthetic potential. The conservative Fisher's test suggests a clear association of CYP705A, CYP708A, CYP702A, CYP716A and ACT IIIa with clade II OSCs [some of them are demonstrated by functional analysis (Fig. 4)]. The phylogenetic methods (PLR and PGLMM) confirm the strong association between CYP705A and clade II OSCs, and suggests additional methods must be used to distinguish the significance of the remaining associations from the result of recently shared ancestry. Such analyses are described further down in this document.

Other associations found with OSC GNs

Among the other significant associations supported by two models are Ferredoxin subdomains (Fer4, Fer4_4, Fer4_7, Fer4_9, Fer4_10), which have been shown to be involved in reduction steps of several primary and specialized metabolic pathways in plants (Hanke & Mulo, 2013), and are strongly associated with clade I OSC GNs; Wall-associated receptor kinases domains (WAK_assoc, GUB_WAK_bind) are also significantly associated with clade I OSC GNs, and have been found to be involved with plant defense and stress responses and regulation of

developmental processes in *A. thaliana* and *O. sativa* (Kanneganti & Gupta, 2008; Hurni *et al.*, 2015); The F-box domain, significantly associated with sterol OSC neighborhoods in both regression models, is involved in regulating plant defense response in *A. thaliana*, *S. lycopersicum* and *N. benthamiana* (van den Burg *et al.*, 2008).

The PLR model also shows significant associations between clade II OSCs and the serine carboxypeptidase domain (Peptidase_S10, SCL acyltransferases). Proteins with this domain are involved in the activation of pathogen defense responses and increased tolerance to abiotic stresses in *O. sativa* and *A. thaliana* (Liu *et al.*, 2008), are essential in the biosynthesis of defense-related metabolites and are part of the avenacin biosynthetic pathway in *A. strigose* (Qi *et al.*, 2004; Mugford *et al.*, 2013), in which it acylates the avenacin triterpene scaffold. The Fisher's exact test also shows that the polyprenyl synthetase domain (polyprenyl_synt), involved in terpenoid biosynthesis in plants (Hsieh *et al.*, 2011), is enriched in clade II OSC neighborhoods.

To reduce the influence of phylogenetic relations in Fisher's exact test, we also used the test in individual genomes rather than in the complete pool of Brassicaceae OSC neighborhoods. This has the disadvantage of losing statistical power by greatly reducing the number of GNs tested, but has the advantage that phylogenetic bias is even more limited (except for those genomes in which whole-genome duplication has recently taken place and most GNs exist in two almost identical copies). This estimate again indicates that the sterol, clade I and clade II OSC genes have distinct genomic neighbourhood associations (Fig. 1**b**; Fig. S1B; Tables S6-7). The Pfam domains associated with OSC genes belonging to the sterol clade are for the most part significantly associated only in a single species (sometimes in two or three, usually closely related species; Fig. S1; Table S7) and, in general, do not have any anticipated roles in specialized metabolism. Interestingly, clade I OSC genes tend to be associated with Pfam domains with functions in electron transfer, gene/chromatin regulation and defence, and these associations were prevalent across multiple diverse Brassicaceae lineages. With regard to enzymes of specialized metabolism, significant associations between Clade I OSC genes and ACT Pfam domains were detected (Fig. S1; Table S7), and there was no evidence that clade I OSCs formed parts of larger biosynthetic gene clusters. The functional significance of these various associations remains to be established. Of direct relevance to the current investigation,

the clade II OSC genes were significantly associated with both CYP and ACT genes, which may encode potential triterpene scaffold-modifying enzymes (Fig. S1; Table S7). Other Pfam domains that were significantly overrepresented in the neighbourhoods of clade II OSC genes include membrane trafficking/transport and gene/chromatin regulation domains. While the latter domains showed associations only within specific species or lineages, the associations with CYP and ACT domains were found across Brassicaceae lineages I and II (Table S7). The CYP association was also evident in the basal Brassicaceae species, *Aethionema arabicum* (Table S7).

CYP705A and ACT IIIa ancestral states reconstruction

In order to investigate when in the evolution of Brassicaceae ACT and CYP genes had been gained or lost from OSC GNs, we performed an initial ancestral state reconstruction analysis [see **Materials and Methods**] on the most prevalent subfamilies found near clade II OSC genes: CYP705A and ACT IIIa. The results of the ancestral state reconstruction, shown as state changes, can be seen in Figure S4A **(left)**, which represent gene recruitment into the neighborhood (0→1), or loss from it (1→0). We illustrated the state changes on the OSC tree generated by FastTree, but we also analyzed the state changes through the RAxML tree, which led us to the same conclusions. The differences between both trees regarding clade II OSCs can be seen in Fig. S2. We found that some CYP705As have likely been independently recruited into closely related neighborhoods without a common ancestor sharing the trait, such as the neighborhoods descendants of the events 2a, 3a and 3b in Fig. S6**a (left)**. Parallel recruitment of ACT IIIa also occurs, as seen in the events 1a, 3a and 3c. To test the robustness of these predictions, we then considered all equivocal states to be 1, which is equivalent to assuming enzymes have been recruited as early as possible while maintaining maximum parsimony; the resulting state changes can be seen in Fig. S6**a (right)**. This changed the predictions for several events. To resolve these uncertainties in ancestral states, we inspected the phylogenetic relationships between the CYP705A and ACT IIIa enzymes identified in the GNs descendant from the aforementioned recruitment events. Genes that were previously identified as part of the same GN are highlighted with the same color in all phylogenetic trees.

Eight GNs descendant from the parallel recruitment events at 1a have an ACT IIIa enzyme; the location of these enzymes in the ACT IIIa phylogenetic tree is highlighted in Fig. S6**c**. The GNs around closely-related *Brara.I04562* and *Bol045822* (highlighted in green) have ACT IIIa

enzymes clustered together in the phylogeny, as shown in Fig. S6**c**, suggesting this enzyme was recruited into an ancestral neighborhood before the split between the *Brassica oleracea* and *Brassica rapa* species. The ACT IIIa enzymes in the second monophyletic group that descend from 1a (highlighted in yellow, gray, purple and pink), which includes the thalianol cluster in *A. thaliana* and *A. lyrata* (yellow), do not cluster in the ACT IIIa phylogenetic tree, suggesting multiple independent recruitments have occurred in this clade. Despite not being in a monophyletic group with the thalianol neighborhoods (yellow), the *Thhalv10027177m* neighbourhood (grey), may descend from the same ancestral neighbourhood, as evidenced by their close distance in both trees. However, it is clear that the *CsLOC104718873*, *CsLOC104732114*, *AL7G40260*, and *AT4G15370* (purple and pink) neighbourhoods have a distinct evolutionary history: the location of the ACT IIIa enzymes in the phylogenetic tree suggests at least two independent recruitments occurred. These two independent recruitments are further supported by reconstructing the ancestral states of ACT IIIa in the OSC tree generated by RAxML: here, the only parsimonious reconstruction is that in which the ACT IIIa enzymes were recruited independently for the two groups. All in all, this advocates the events at 1a to be more likely than that at 1b, and indicates that the ACT IIIa recruitments of the second monophyletic group (yellow, grey, purple and pink) occurred as two parallel independent recruitments at less basal branches.

Six GNs descendant from the 2a/b events have a CYP705A enzyme; the location of these enzymes in the CYP705A phylogenetic tree is highlighted in Fig. S6**b**. The thalianol neighborhoods (yellow) both have a single CYP705A, which are clustered together in the CYP705A tree. The remaining neighborhoods (purple and pink) have multiple CYP705A enzymes: a monophyletic group closely related to the thalianol CYP705As, a paraphyletic group of three enzymes, and two phylogenetically distantly related additional enzymes that only the *AT4G15370* (arabidiol/baruol - pink) GN has recruited. This suggests four distinct CYP705A recruitment events in this clade: the recruitment of the thalianol CYP705A at 2b, an additional recruitment after a duplication event from which the present thalianol clusters diverged (at the lower 2a event), and two recent independent recruitments into the *AT4G15370* (arabidiol/baruol - pink) GN.

Lastly, four GNs descend from the events 3a/b/c/d; the location of their CYP705A and ACT IIIa enzymes are highlighted in Figs. S4**b-c**. The events 3a and 3b indicate independent parallel recruitment of three CYP705A enzymes, however, they are clustered closely in the CYP705A phylogenetic tree, indicating this enzyme was recruited into an ancestral GN from which all three descend, perhaps at a branch more basal than the 3d event already suggests. In a similar manner, the ACT IIIa enzymes identified in these neighborhoods cluster close to each other in the ACT IIIa phylogenetic tree, which indicates that the recruitment of these ACT IIIa enzymes occurred once in a genomic neighborhood of a common ancestor, unlike both ancestral reconstructions indicate.

Integrating this together, we reached a consensus ancestral reconstruction, which can be seen in Fig. 1**a**. This tree reconstruction evidences at least three independent recruitment events of distinct CYP705A genes and at least three independent recruitment events of distinct ACT_IIIa genes into clade II OSC GNs through the Brassicaceae evolutionary history. This indicates that the similarity in domain content of many OSC GNs is not due to an ancestral locus conserved through speciation, but rather due to independent assembly, which has resulted in distinct specialized metabolic pathways encoded in BGCs: the thalianol and tirucallol pathways (in lineage I) and euphol pathways (in lineage II).

Analysis of GNs in light of the evolutionary history of Brassicaceae

As a result of the numerous ancient WGDs in Brassicaceae, multiple chromosomal regions in the *A. thaliana* genome are syntenic with each other. These "conserved" blocks have been identified in previous studies (Freeling *et al.*, 2007) and are defined by "anchor genes", which each have high sequence identity with their homolog in the syntenic region. Additionally, identifying these syntenic blocks defines more dynamic regions that no longer retain any identifiable synteny within the genome. *A. thaliana* has ten OSC GNs: two around clade I OSCs, six around clade II OSCs and two around sterol OSCs. Of these, four are in dynamic regions: three out of the six clade II GNs (AT5G48010 [the thalianol BGC], AT5G42600 [the marneral BGC] and AT3G29255) and one sterol GN (AT2G07050).

We compared the remaining GNs with their respective syntenic region as defined by the anchor genes around each GN by examining their protein sequence alignments and domain content [see

Materials and Methods]. The results of this analysis, displayed in Fig. S9, show that while the remaining three clade II OSC GNs are not located in dynamic chromosomal regions, they are located in highly dynamic 'pockets' within their syntenic blocks.

The characterized Arabidiol/Baruol BGC (Castillo *et al.*, 2013) is contained within the AT4G15340 GN (Fig. S9**a**), fully flanked by the anchor genes *AT4G15280* and *AT4G15410*. The region syntenic to this GN, located between the genes *AT3G21660* and *AT3G21750*, shares no protein domain content with the Arabidiol/Baruol GN and has very low sequence identity.

The AT1G78500 clade II OSC GN (Fig. S9**b**) contains a few anchor genes: *AT1G78460*, *AT1G78510*, *AT1G78540* and *AT1G78550*. Its syntenic region is flanked by *AT1G16700* and *AT1G16820*, and only the aforementioned anchor genes and some repeats show any synteny between the two loci. Notably, this GN involves one *CYP708A* (*AT1G78490*), which resides directly next to the OSC within a window of 5 genes without synteny. Interestingly, the syntenic region also contains a gene that encodes a P450, *AT1G17060*; however, the enzyme expressed belongs to a different subfamily (CYP72C1). The OSC phylogeny (Fig. 1) shows this *A. thaliana* OSC clusters with similar genes in *C. sativa*, *A. lyrata* and *A. halleri*, and their respective GNs contain a CYP708A, with the exception of *A. halleri*, which is located on a short scaffold; notably, the *A. thaliana* and *A. lyrata* CYP708As also cluster together in the P450 phylogeny along with an *A. halleri* CYP708A that resides in a short two-genes scaffold. Altogether, this indicates that the genomic structure of the neighboring OSC-P450 pair was inherited from an early Brassicaceae lineage I ancestor.

The AT5G36150 clade II OSC GN (Fig. S9**c**), which encompasses the characterized tirucalla pathway (Boutanaev *et al.*, 2015), is flanked by the two anchor genes, *AT5G36100*, and *AT5G36230*. This region encompasses 12 genes without any synteny or shared domain content with its sister region in chromosome 1, flanked by the anchor genes *AT1G65090* and *AT1G65220*. None of the GNs around the OSCs clustered with the tirucalla OSC in the OSC phylogeny show any similarity with this GN, indicating the tirucalla BGC is likely a very recent assembly, or has been lost in the other species.

The two clade I OSC GNs located in WGD-derived syntenic blocks of the *A. thaliana* genome show high architectural and sequence similarities with GNs in other lineage I species, and one of them shows high similarity to its sister region within the same genome. This suggests clade I OSC GNs are much less dynamic than clade II OSCs GNs. On the other hand, sterol OSC GNs appear to fall somewhere in the middle, with the only one located in a WGD-derived syntenic block showing no similarity with its syntenic locus, but some similarity to a GN in *A. lyrata*.

Specifically, the AT1G66960 clade I OSC GN (Fig. S9**d**) is flanked by the anchor genes *AT1G66910* and *AT1G67035*. Notably, this region involves many Pkinase tandem repeats that show relatively high sequence identity with genes in the syntenic region, flanked by *AT5G38240* and *AT5G38300*. The clade I OSC in the GN and its two immediate neighbors, however, show no synteny with the sister region. This OSC clusters in a group of OSCs for which the phylogenetic topology closely resembles the species phylogeny (a clade of OSCs from lineage I species and a clade of OSCs from lineage II species). The corresponding GNs in the lineage I species *A. halleri* and *A. lyrata* have the same protein domain content and high average amino acid identity (70% and 67% respectively), suggesting this GN has been inherited from a Brassicaceae lineage I common ancestor.

The AT1G78950 clade I OSC GN (Fig. S9**e**) is defined by anchor genes *AT1G78900* and *AT1G79010*, and its syntenic region is between the genes *AT1G16700* and *AT1G16820*. Notably, this GN contains an ACT IIIa gene (*AT1G78990*), which is located in the same window of non-syntenic genes as the OSCs in this GN (*AT1G18950-970*), defined by the anchor genes *AT1G79000* and *AT1G78940*. Interestingly, in the OSC phylogeny (Fig. 1), this *A. thaliana* gene clusters with similar OSCs in *C. sativa*, *A. alpina*, *B. stricta* and *A. lyrata*, which have ACT IIIb within their GNs that also cluster together in the ACT phylogeny (Fig. S1). This indicates that this GN has also been inherited from an early Brassicaceae lineage I ancestor.

The AT3G45130 sterol OSC GN (Fig. S9**f**) is flanked by the anchor genes *AT3G45060* and *AT3G45210*. The region syntenic to this GN is located between *AT5G60680* and *AT5G60770*. Notably, there is no protein domain content shared among the two regions, with the only gene showing any sequence identity being *AT3G45090*, another anchor gene within the GN. This OSC is phylogenetically clustered close to the *A. lyrata* OSC *AL5G23630*, and the two GNs share

around half of the protein domain content (Jaccard index = 0.53), and relatively high sequence similarity (DSS = 0.56 and average amino acid identity = 83%).

To further explore the evolutionary history of the *A. thaliana* OSC GNs, we mapped the OSCs within the Ancestral Crucifer Karyotype (ACK) genomic blocks of Brassicaceae. The 24 genomic blocks of the ACK are characterized by having retained high conservation among Brassicaceae species, and have been identified in *A. thaliana*, *A. lyrata*, *C. rubella* and *A. alpine* (Murat *et al.*, 2015; Lysak *et al.*, 2016). Using the OSC phylogeny, we identified the eight *A. lyrata* OSCs closely related to eight *A. thaliana* GNs, five *A. alpina* OSCs closely related to four *A. thaliana* GNs, and two *C. rubella* OSCs closely related to two *A. thaliana* GNs. We then mapped these GNs within the ACK genomic blocks (Table S9).

We found that the majority of homologous OSCs reside in the same ACK block: The AT2G07050 and AT3G29255 GNs are located within the G and L genomic blocks respectively, and each have one closely related OSC in *A. lyrata* and *C. rubella* that can be found in the same corresponding genomic blocks; similarly, the Arabidiol/Baruol and Thalianol pathway GNs are located in the T and W genomic blocks respectively, and each have a homologous OSC in *A. lyrata* that is also located within the same genomic blocks; the AT3G45130 GN, located in the M genomic block, has one homologous OSC in *A. lyrata*, also located in the M genomic block, and two in *A. alpina*: AALP_AA5G0337300 and AALP_AA5G078800; only the latter is also located in the M genomic block. Lastly, the three *A. thaliana* GNs located in the E genomic block (AT1G66960, AT1G78500 and AT1G78950) each have one counterpart in *A. lyrata* and *A. alpina*, and only one of the *A. alpina* GNs (AALP_AA3G344300) is not located within the E genomic block.

The topology of the phylogeny for these OSC genes and their similar chromosomal location within the ACK genomic model allow us to conclude there is an orthologous relationship between them. Interestingly, two of the monophyletic and likely orthologous pairs of OSCs discussed above do not reside in the same genomic block, indicating translocations have likely taken place. The *A. alpina* OSC *AALP_AA3G344300* is located in the H genomic block, while its closely related OSCs in *A. lyrata* and *A. thaliana* are located in the E genomic block. This suggest that either the *A. alpina* OSC was translocated from the E to the H genomic block at

some point after *A. alpina*'s divergence, or alternatively, that the corresponding gene in an *A. lyrata-A. thaliana* ancestor has been translocated from the H to the E genomic block. On the other hand, the direction of the second translocation event is clearer: AALP_AA5G0337300 and AALP_AA5G078800 have 93% protein sequence identity, suggesting they are likely the result of a recent duplication event in *A. alpina*. Because only *AALP_AA5G078800* is in the same genomic block as its homologs in *A. thaliana* and *A. lyrata*, *AALP_AA5G0337300* was likely translocated from the M to the K-L genomic block, as the alternative scenario would require at least two separate yet similar translocation events: one in *A. alpina* and one in an *A. lyrata-A. thaliana* ancestor, and both from the K-L to the M genomic block.

Notably, the *C. rubella* tirucallol BGC we characterized within this study includes the OSC *Carubv10016727m*, which is phylogenetically clustered with *AL5G19180* from *A. lyrata* (71% protein sequence identity) and *AT3G29255* from *A. thaliana* (70% protein sequence identity), both of which are located in the L genomic block. While the GNs around the two Arabidopsis OSCs have some mutual similarities in protein domain content (Pfams: PPR, DUF179, zf-RING-like), they share no similarity with the *C. rubella* GN. On the other hand, both the *B. stricta* OSC Bostr.0556s0559 (highlighted in teal in Fig. S6**a**) that is clustered with the Arabidopsis OSCs in the phylogenetic tree, and the *C. sativa* OSCs that are clustered with the Tirucallol OSC (CsLOC104715121, CsLOC104773008 and CsLOC104780146) (also highlighted in teal in Fig. S6a), have GNs which show high similarity with the tirucallol BGC in protein domain content and sequence identity (highlighted in teal in Figs. S4**b-c**). Recent phylogenetic analyses of the Brassicaceae suggest that *A. thaliana* does not descend from the most recent common ancestor of *B. stricta*, *C. sativa* and *C. rubella* (Nikolov *et al.*, 2019); therefore, the high similarity between these GNs and their conserved chromosomal location within the same ACK genomic model suggest that the tirucallol GN, and its homologs, have been inherited from a GN that was ancestral to *Boechera*, *Camelina* and *Capsella*, but assembled after the split from *Arabidopsis*.

In conclusion, no Brassicaceae OSC GN appears to be conserved through the ancient Brassicaceae WGD events, and although some synteny can be found among GNs of different species, most similarity is constrained exclusively within lineage I or lineage II, which altogether highlights the dynamic nature of OSC GNs. The Brassicaceae OSC genes, however, appear to have rather conserved chromosomal locations, suggesting the assembly of the GNs that result in

functional pathways likely occur through the translocation of genes encoding tailoring enzymes. Our research shows that the assembly of these GNs often results in similar genomic architectures despite having a distinct evolutionary history.

4. Supplementary Reference

Beilstein MA, Nagalingum NS, Clements MD, Manchester SR, Mathews S. **2010**. Dated molecular phylogenies indicate a Miocene origin for *Arabidopsis thaliana*. *Proceedings of the National Academy of Sciences* **107**: 18724 LP – 18728.

Boutanaev AM, Moses T, Zi J, Nelson DR, Mugford ST, Peters RJ, Osbourn A. **2015**. Investigation of terpene diversification across multiple sequenced plant genomes. *Proceedings of the National Academy of Sciences USA* **112**: E81-8.

van den Burg HA, Tsitsigiannis DI, Rowland O, Lo J, Rallapalli G, Maclean D, Takken FLW, Jones JDG. **2008**. The F-box protein ACRE189/ACIF1 regulates cell death and defense responses activated during pathogen recognition in tobacco and tomato. *The Plant cell* **20**: 697–719.

Castillo DA, Kolesnikova MD, Matsuda SPT. **2013**. An effective strategy for exploring unknown metabolic pathways by genome mining. *Journal of the American Chemical Society* **135**: 5885–5894.

Edgar RC. **2004**. MUSCLE: multiple sequence alignment with high accuracy and high throughput. *Nucleic Acids Research* **32**: 1792–1797.

Field B, Osbourn AE. **2008**. Metabolic diversification—independent assembly of operon-like gene clusters in different plants. *Science* **320**: 543 LP – 547.

Freeling M, Rapaka L, Lyons E, Pedersen B, Thomas BC. **2007**. G-boxes, bigfoot genes, and environmental response: characterization of intragenomic conserved noncoding sequences in Arabidopsis. *The Plant Cell* **19**: 1441–57.

Gotoh O. **1992**. Substrate recognition sites in cytochrome P450 family 2 (CYP2) proteins inferred from comparative analyses of amino acid and coding nucleotide sequences. *Journal of Biological Chemistry* **267**: 83–90.

Graber S. **2013**. Phylogenetic comparative methods for discrete responses in evolutionary biology (Master thesis, University of Zurich, Faculty of Science.).

Hanke G, Mulo P. **2013**. Plant type ferredoxins and ferredoxin-dependent metabolism. *Plant, cell & environment* **36**: 1071–84.

Hsieh F-L, Chang T-H, Ko T-P, Wang AH-J. **2011**. Structure and mechanism of an Arabidopsis medium/long-chain-length prenyl pyrophosphate synthase. *Plant physiology* **155**: 1079–90.

Hurni S, Scheuermann D, Krattinger SG, Kessel B, Wicker T, Herren G, Fitze MN, Breen J, Presterl T, Ouzunova M, *et al.* **2015**. The maize disease resistance gene Htn1 against northern corn leaf blight encodes a wall-associated receptor-like kinase. *Proceedings of the National Academy of Sciences USA* **112**: 8780–5.

Kanneganti V, Gupta AK. **2008**. Wall associated kinases from plants - an overview. *Physiology and molecular biology of plants* **14**: 109–18.

Leong YW, Harrison LJ. **1999**. (20R,23E)-eupha-8,23-diene-3β,25-diol from Tripetalum cymosum. *Phytochemistry* **50**: 849–857.

Liu H, Wang X, Zhang H, Yang Y, Ge X, Song F. **2008**. A rice serine carboxypeptidase-like gene OsBISCPL1 is involved in regulation of defense responses against biotic and oxidative stress. *Gene* **420**: 57–65.

Lysak MA, Mandáková T, Schranz ME. **2016**. Comparative paleogenomics of crucifers: ancestral genomic blocks revisited. *Current Opinion in Plant Biology* **30**: 108–15.

Morlacchi P, Wilson WK, Xiong Q, Bhaduri A, Sttivend D, Kolesnikova MD, Matsuda SPT. **2009**. Product profile of PEN3: the last unexamined oxidosqualene cyclase in *Arabidopsis thaliana*. *Organic Letters* **11**: 2627–2630.

Mugford ST, Louveau T, Melton R, Qi X, Bakht S, Hill L, Tsurushima T, Honkanen S, Rosser SJ, Lomonossoff GP, *et al.* **2013**. Modularity of plant metabolic gene clusters: a trio of linked genes that are collectively required for acylation of triterpenes in oat. *The Plant cell* **25**: 1078–92.

Murat F, Louis A, Maumus F, Armero A, Cooke R, Quesneville H, Crollius HR, Salse J. **2015**. Understanding Brassicaceae evolution through ancestral genome reconstruction. *Genome Biology* **16**: 262.

Nikolov LA, Shushkov P, Nevado B, Gan X, Al-Shehbaz IA, Filatov D, Bailey CD, Tsiantis

M. **2019**. Resolving the backbone of the Brassicaceae phylogeny for investigating trait diversity. *New Phytologist* **222**: 1638–1651.

Nishitoba T, Oda K, Sato H, Sakamura S. **1988**. Novel triterpenoids from the fungus Ganoderma lucidum. *Agricultural and Biological Chemistry* **52**: 367–372.

Qi X, Bakht S, Leggett M, Maxwell C, Melton R, Osbourn A. **2004**. A gene cluster for secondary metabolism in oat: implications for the evolution of metabolic diversity in plants. *Proceedings of the National Academy of Sciences USA* **101**: 8233–8.
